# Selective attraction to shorter muzzles in dogs is a hidden driver of the brachycephalic welfare crisis

**DOI:** 10.1038/s41598-025-16562-9

**Published:** 2025-10-06

**Authors:** Zsófia Bognár, Yuri Kawaguchi, Koyo Nakamura, Enikő Kubinyi

**Affiliations:** 1 MTA-ELTE Lendület “Momentum” Companion Animal Research Group, Budapest, Hungary; 2 Department of Ethology, ELTE Eötvös Loránd University, Budapest, 1117 Hungary; 3 School of Social Sciences, Nottingham Trent University, Nottingham, England; 4 Faculty of Science and Engineering, Waseda University, Tokyo, Japan; 5 Japan Society for the Promotion of Science (JSPS), Tokyo, Japan; 6 Faculty of Psychology, Department of Cognition, Emotion, and Methods in Psychology, University of Vienna, Vienna, Austria; 7 ELTE NAP Canine Brain Research Group, Budapest, Hungary

**Keywords:** Dog, Brachycephalism, Muzzle length, Image manipulation, Personality, Ideal dog, Human behaviour, Animal behaviour, Patient education

## Abstract

Modern dog breeds show a trend towards shorter muzzles in the Western world. Using an international online questionnaire (*N* = 780), we examined how muzzle length, as the sole varying feature, influenced visual preference for dogs, using digitally morphed images of the same individuals. We investigated how preferences for shorter muzzles were influenced by demographics, personality, pre-existing attitudes towards brachycephalic dogs, awareness of health issues, and desired dog traits. Shorter muzzles were generally preferred. Associating more health problems with brachycephaly was negatively associated with preference for shorter muzzles, except among those with positive attitudes towards brachycephalic dogs. Preference was positively associated with positive attitudes towards brachycephalic dogs, older age, conscientiousness, extraversion, and appreciation of aesthetics; and negatively associated with dog-related professional expertise, openness, and a liking for control in dog keeping. Among respondents without prior attitudes, preference was positively linked to viewing the ideal dog as childlike, and negatively to valuing a humorous personality in the ideal dog. Our results show how age, dog-related profession, personality, health awareness, and attitudes towards brachycephalic dogs and the ideal dog intersect to shape preferences for shorter muzzle length. Our findings support the notion that individuals preferring shorter muzzles may prioritise aesthetic traits over health concerns.

**Supplementary Information:**

The online version contains supplementary material available at 10.1038/s41598-025-16562-9.

## Introduction

Modern dog breeds exhibit considerable cranial morphological variation, both between and within breeds^1–3^with a general trend towards decreasing skull length observed in the Western world. This reduction is driven by the growing popularity of extremely brachycephalic (short-headed) breeds^4–8^as well as by a generational shortening of skulls within individual breeds^9^.

A shorter muzzle in dogs originally emerged either through selection for stronger bite force or as a consequence of selection for smaller body size. Humans appear to have an inherent preference for infant-like facial appearance, such as a flatter face, larger foreheads, and bigger eyes – characteristics that are responsible for triggering the ‘baby schema effect’ and eliciting increased attention and a willingness to care for individuals showing a ‘baby schema’^10–14^. A shorter head shape in dogs is associated with a more infant-like facial appearance^15^, which likely contributes to the increasing popularity of brachycephalic breeds and has driven a trend towards progressively shorter average muzzle lengths in modern dog populations^4–7,9^. This trend resembles a runaway process^16^, where aesthetic preferences for infant-like features may have intensified selection for increasingly extreme short-headed conformations. One possible explanation for this growing popularity is the human innate predisposition for caregiving behaviour. Given that only the minority adults (e.g., 13% in Hungary) are actively involved in caring for children, an ecological niche for caregiving may have opened, with dogs—particularly those displaying infant-like features—fulfilling this role^17^.

Extensive evidence suggests that extreme skull shortening can lead to a wide range of diverse and severe health problems^18,19^. The most common and well-known disorder associated with the shortening of the skull is the so-called brachycephalic obstructive airway syndrome (BOAS)^20^. This respiratory disorder arises from anatomical abnormalities associated with skull shortening in the upper airway of extremely brachycephalic dogs, such as congenital, inherited malformations of the nostrils, nasopharynx, and soft palate^21–23^. BOAS leads to chronic dyspnoea, sleep apnoea due to breathing difficulties, gastrointestinal disorders due to the excessive respiratory effort (such as reflux, hiatal hernia, oesophagitis), obesity due to exercise intolerance, and heat stroke due to heat intolerance^24–28^. The prevalence of BOAS is notably high in extremely brachycephalic dogs, with estimates suggesting that only around 10% are unaffected^22^. The early mortality of brachycephalic dogs is largely attributed to BOAS^19,29–31^.

Extremely shortened skull is also associated with malformed, shallow eye sockets, which results in the vulnerable ‘bulging’ eyes. This, in turn, leads to a range of ophthalmological disorders due to impaired eyelid function and reduced ocular protection, such as corneal ulcers, dry eye, and proptosis^32–34^. The nasal skin folds of these dogs may also predispose them to ocular trauma and, due to the moist environment within the folds, to infectious skin diseases^35,36^. The shortened skulls of these dogs also lead to dental abnormalities due to overcrowded and rotated teeth^19^. Congenital brain disorders, such as hydrocephalus and Chiari-like malformation, are also common in extremely brachycephalic dogs^19,37^. Beyond disorders linked to extreme skull shortening, these dogs also show a high predisposition to vertebral and spinal malformations^38–40^. Due to hereditary anatomical abnormalities and breathing difficulties, reproductive problems (dystocia, natural mating inability) are also common in extreme brachycephalic dog breeds^19,41,42^.

Health issues can also negatively affect the dog-human relationship. Unsurprisingly, brain disorders can lead to aggression or other behaviour problems^19,43^. However, not only neurological conditions but also pain itself can cause aggression and problem behaviours in dogs^44,45^, including those arising from musculoskeletal, gastrointestinal, or dermatological disorders^46^. Indeed, aggression is a common reason why owners of French Bulldogs seek veterinary assistance^47^. This may also be due to their impaired facial expressions, which can lead to misinterpretation of their behaviour by humans^48,49^. Undesirable behaviour, particularly aggression towards familiar humans or animals, is a common reason for relinquishment or even euthanasia^50–58^. However, the presence of health problems appears to have a positive effect on the attachment between owners and their brachycephalic dogs^59^. This may be due to increased caregiving behaviours from the owner, which can contribute to the development of a strong bond^60^, as well as the influence of the dogs’ ‘baby schema’ features^10–14^. However, it is likely that owners whose relationships with their dogs have deteriorated due to behaviour problems resulting from health issues—and who have subsequently relinquished or euthanised their dogs—do not participate in such studies, potentially introducing bias into the results.

If the extremely short head shape has such serious health consequences, how can it persist? Because of the runaway process, traits that would otherwise negatively impact an individual’s survival and reproductive success can spread through the population^16^. In small, short-headed dog breeds, the expected lifespan is the shortest^19,30,31^, and their reproductive success without intervention is also extremely low^41,42,61^. The runaway process would theoretically reach a standstill if the cost of carrying the trait became too high in terms of survival and reproductive success^16^. However, humans successfully compensate for the detrimental effects through veterinary interventions, both by increasing lifespan with plastic surgeries and by improving reproductive success through artificial insemination and Caesarean sections^19,41,42,61,62^.

The growing popularity of the extremely short head shape, combined with its association with severe health problems, has led to a dog welfare crisis^19^. As a response, some countries have already made attempts to regulate or ban brachycephalic dog breeds^63–68^. To better understand and address this welfare crisis, further research is needed to identify the factors contributing to the trend towards decreasing skull length in the modern dog population in the Western world.

When investigating the pre-purchase motivations of owners of popular dog breeds—that is, the factors influencing their breed choice—it was found that appearance and the perception of the breed as a ‘good companion’ were more influential for those choosing brachycephalic breeds than for those selecting non-brachycephalic breeds^69^. In contrast, the perception of the breed as being ‘generally healthy’ and having a long life expectancy was less influential among brachycephalic dog owners compared to non-brachycephalic dog owners^69^. Other studies examining pre-purchase motivations reported that the breed’s appearance, behaviour and personality were particularly valued by owners of extreme brachycephalic dogs^59,70^. When brachycephalic dog owners were asked why they would recommend their breed to prospective dog owners, they cited the breed’s positive behavioural traits as a companion dog, its ‘humorous’ personality, and its ‘lazy’ temperament^71^.

The appeal of extreme brachycephalic dog breeds’ appearance may also be attributed to owners’ preference for individuals with more extremely shortened muzzle, even within brachycephalic breeds^72^, suggesting that muzzle length plays a central role in aesthetic preferences. Consistent with this, owners of extremely brachycephalic dogs are more likely to consider a flat face a desirable trait compared to owners of other dog types^73^. Furthermore, extremely short muzzles (that is, extreme brachycephaly) may be particularly appealing to individuals who are motivated by external recognition^72^. However, previous studies have not directly manipulated muzzle length or controlled for confounding factors, leaving it unclear whether shorter muzzles truly influence people’s preferences.

Public attitudes towards brachycephalic dog breeds appear to be influenced by various demographic factors and human personality traits^74^. For instance, younger individuals and women tend to have more positive attitudes towards these dogs^74^, possibly due to their greater sensitivity to the baby schema effect^75–78^. However, other studies have reported that brachycephalic dog breeds are predominantly owned by older adults^8^, likely due to the perception that these dogs are easier to manage as a result of their lower exercise requirements^71,73^. Additionally, parents are also more likely to have positive attitudes towards brachycephalic dogs^74^, which may be explained by a sensitivity to infantile features^78^, or the perception that such dogs—due to their small size, presumed child-friendliness, and low activity levels—are well suited for family life^71,73^.

Conversely, individuals with higher levels of education in general, as well as those with animal- or dog-related professional expertise (e.g., veterinarians, dog trainers), are more likely to have a negative attitude towards brachycephalic dogs^74^, brachycephalic cats^79^, and brachycephalic rabbits^80^. This is likely attributable to their awareness of the health and welfare issues associated with extreme brachycephaly. Interestingly, however, brachycephalic dog enthusiasts’ knowledge about the health problems of these dogs does not influence their positive attitudes towards them^74^. In line with this, brachycephalic dog owners normalise and accept the health problems as inherent traits of brachycephalic dogs^70,81–85^.

When examining the Big Five personality traits, individuals with negative attitudes towards brachycephalic dogs have been found to score lower in agreeableness and conscientiousness^74^. Both traits have been shown to predict preferences that align with sociocultural norms^86^, such as the increasing popularity of extremely brachycephalic dog breeds in recent years^4–8^. Individuals low in agreeableness and conscientiousness may be more inclined to intentionally deviate from social norms. Furthermore, these personality traits have been linked to how individuals emotionally interpret static images of dogs^87^, suggesting that those lower in agreeableness and conscientiousness may be more prone to attributing negative emotional states to brachycephalic dogs. However, the dogs’ head shape itself was not found to significantly influence emotional attribution^87^.

### Aims and research questions

Numerous factors influence the choice of a companion animal, including its breed and appearance. While several factors associated with the choice of brachycephalic dog breeds have already been identified, gaps remain in our understanding of whether these factors merely influence breed choice or also affect the preference for shorter muzzle length in dogs.

**Fig. 1:**
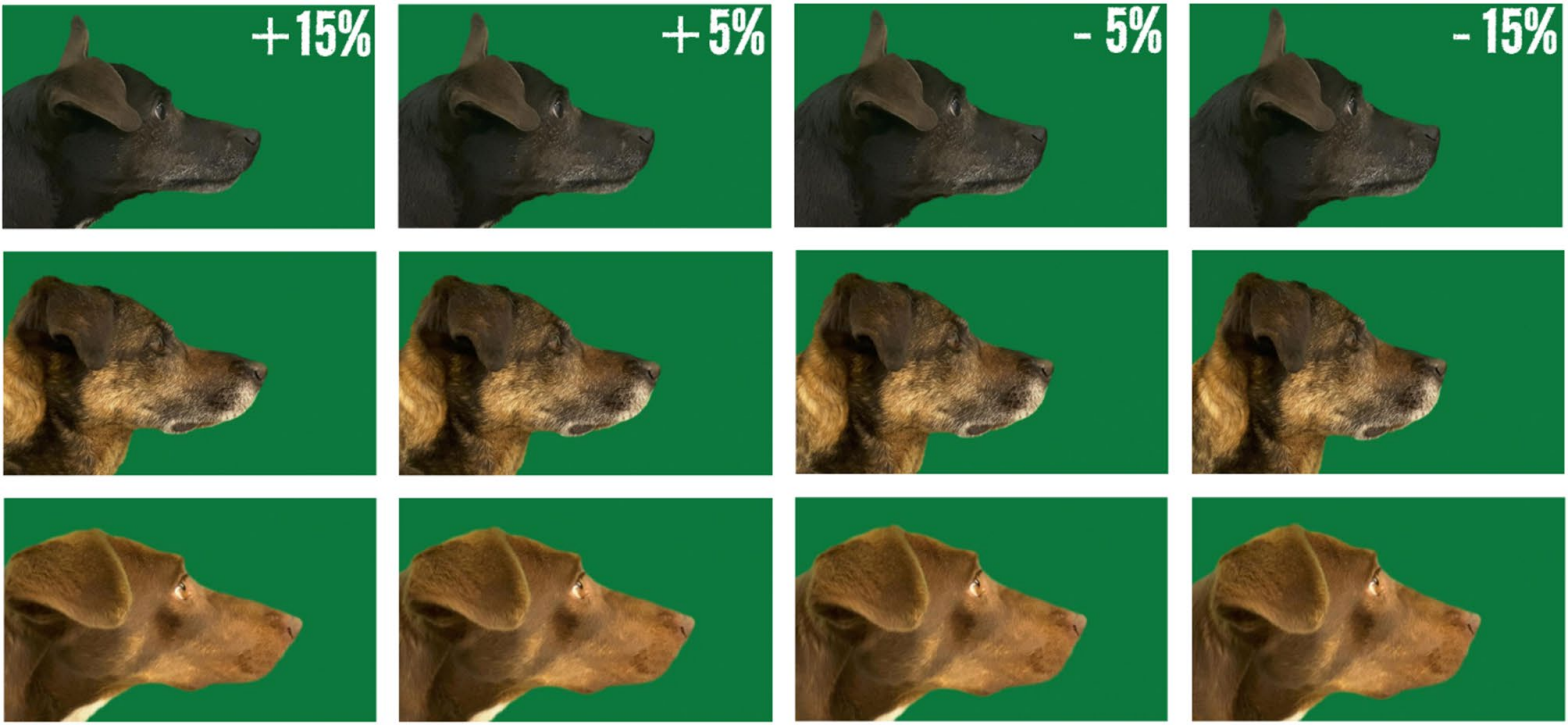
Examples of muzzle length manipulations. We presented four photos of each dog, each depicted with four muzzle lengths [much longer (+ 15%), slightly longer (+ 5%), slightly shorter (−5%), and much shorter (−15%), than the original]. Respondents were asked which photo they liked more. The full set of photo stimuli is available in Supplementary Table 1.

Our study aimed to investigate the factors influencing preferences for muzzle length when muzzle length is the only visible difference between individual dogs. To achieve this, we employed a different methodological approach: instead of asking breed-related questions, we assessed participants’ preferences for muzzle length using a set of digitally morphed dog profile photos. Each image depicted the same individual dog with varying muzzle lengths, thereby excluding other factors (e.g., coat colour or length, ear shape) that might influence preferences (see examples in Fig. 1; full set in Supplementary Table 1). Potential correlations with previously identified factors associated with attitudes towards brachycephalic dog breeds were further analysed.

Given that some individuals hold negative attitudes towards brachycephalic dog breeds, we assumed that shorter muzzle length may not be aesthetically preferred by everyone, and that pre-existing attitudes might also influence preferences for muzzle length. Therefore, we expected that although similarities might be observed across attitude groups in terms of factors influencing muzzle length preference, distinct patterns would also emerge among individuals with pre-existing positive or negative attitudes.

We hypothesised that younger individuals and women would prefer shorter muzzle lengths, whereas individuals with higher levels of education and those with animal- or dog-related professional expertise (e.g., veterinarians, dog trainers) would be less likely to do so. We also assumed that the more health issues respondents associated with brachycephalism, the less likely they would be to prefer shorter muzzle lengths.

We expected that both the level and the field of education play a role in shaping people’s preferences for muzzle length, as they may affect the ability to critically assess and interpret information about the health and welfare problems of brachycephalic dogs—information to which people are increasingly exposed via social media, partly as a result of awareness campaigns^88–90^. Specifically, we assumed that individuals with a background in health-related fields—whether in human or veterinary medicine—are better equipped to comprehend the health implications of exaggerated conformations. In addition, we assumed that a background in the natural sciences fosters a deeper understanding of natural phenomena and their interrelationships, thereby enhancing the ability to grasp the multifaceted consequences of extreme brachycephaly on animal welfare.

We also expected that individuals who score lower in the Big Five personality traits of agreeableness and conscientiousness would be less likely to prefer shorter muzzle length, as previously found in relation to pre-existing attitudes towards brachycephalic dogs^74^.

We also explored whether respondents’ relationship with children influences their preference for shorter muzzle length, given the potential link between declining human birth rates and the rising popularity of small-sized, brachycephalic dog breeds^17^. We assumed that having less contact with children would be associated with a greater likelihood of preferring shorter muzzle lengths.

Furthermore, dog lovers differ in how they perceive and relate to dogs—whether they regard them as quasi-human companions, such as family members, children, or close friends, as affectionate pets, or merely as utilitarian animals^91–93^. Individuals may also vary in how they conceptualise their ideal dog. Two studies examining the characteristics of an ideal companion dog found that, for the majority of respondents, it was important that the dog be safe with children, physically healthy, fully housetrained, affectionate towards its owner, and live to at least 10 years of age^94,95^. Preferences for specific breeds or dog types may also be influenced by what individuals find most rewarding about dog ownership^93^. In the present study, we examined whether preferences for shorter muzzle length are associated with people’s expectations of a hypothetical ideal dog—specifically, its traits and its perceived role within the respondent’s social network—as well as with the aspects of dog ownership they consider most rewarding. We hypothesised that perceiving dogs as family members or as children, as well as valuing a humorous personality, low exercise needs, and aesthetic appearance in a hypothetical dog, would increase the likelihood of preferring shorter muzzle lengths.

We also hypothesised that individuals with pre-existing positive attitudes towards brachycephalic dogs^74^ would prefer shorter muzzle lengths, whereas those with pre-existing negative attitudes would be less likely to do so. To identify potential differences in patterns between individuals with pre-existing positive or negative attitudes, associations with the aforementioned factors were also examined separately within each attitude group.

Taken together, we aimed to examine the potential associations between a preference for shorter muzzle length and: (1) various human characteristics, including demographic and personality traits; (2) the qualities people seek in dogs, such as the attributed role of a hypothetical ideal companion dog, the desired traits, and perceived rewards of dog ownership; and (3) pre-existing attitudes towards brachycephalic dogs, as well as knowledge of the health problems associated with brachycephaly.

## Methods

### Ethical statement

The United Ethical Review Committee for Research in Psychology (EPKEB) approved and accepted the experimental protocol (Ref. no.: 2022-99). All experiments were performed in accordance with relevant guidelines and regulations, and informed consent was obtained from all participants.

### Participants

The questionnaire was developed to reach an international convenience sample. It was advertised on Facebook on the International (https://www.facebook.com/FamilyDogProject) and Hungarian Family Dog Project pages (https://www.facebook.com/CsaladiKutyaProgram), as well as in the public Hungarian Facebook group dedicated to dog ethology and handled by researchers of the Family Dog Project (https://www.facebook.com/groups/kutyaetologia). Family Dog Project is an umbrella term for dog-related research groups based at the Department of Ethology, Eötvös Loránd University and the HUN-REN KPI in Budapest, Hungary (https://ethology.elte.hu/Family_Dog_Project). Family Dog Project started 30 years ago and is the world’s leading dog behaviour research group^96^.

The questionnaire was open between November 2022 and February 2024. The questionnaire was posted once in November 2022 on pages belonging to the Family Dog Project. Due to the low numbers of brachycephalic dog enthusiasts, the questionnaire was posted in Hungarian and international Facebook groups of brachycephalic dog breeds after permission by the administrators was granted (once per groups). As an incentive, upon completing the questionnaire, respondents could check their results of the Big Five questionnaire using a unique code, which was optionally generated at the end of the questionnaire.

Participants were required to be over the age of 18; beyond that, anyone could complete the questionnaire, regardless of their nationality or whether they currently own or have previously owned a dog. Although the questionnaire was distributed internationally, it was available only in English and Hungarian, thus a bias towards English and Hungarian speaking respondents was expected. The majority of respondents were from the country of origin of the study, which is common in similar international studies^48,84^.

A total of 780 participants took part in the study (436 participants in the Hungarian version, 344 in the English version). As 97.8% of respondents (*N* = 763) held a nationality from the Western world, our analysis focused exclusively on this group. The modern concept of the Western world – also referred to as the ‘Latin West’ – is based on cultural rather than geographical factors and therefore includes countries shaped by Western European culture (https://worldpopulationreview.com/country-rankings/western-countries). See Table 1 for the list of nationalities included or excluded in the analysis.

**Table 1:** Nationalities included or excluded in the analysis according to their classification as ‘Latin West’ countries.

Nationalities outside of the ‘Latin West’ countries (nationalities excluded from the analysis)		Number of respondents (% of total sample)	
**1**	Russian	*N* = 3 (0.4%)	
**2**	Singaporean	*N* = 2 (0.3%)	
	South African	*N* = 2 (0.3%)	
	Taiwanese	*N* = 2 (0.3%)	
**3**	Caribbean	*N* = 1 (0.1%)	
	Israeli	*N* = 1 (0.1%)	
	Japan	*N* = 1 (0.1%)	
	Romanian	*N* = 1 (0.1%)	
	South Korean	*N* = 1 (0.1%)	
	Turkish	*N* = 1 (0.1%)	
	Ukrainian	*N* = 1 (0.1%)	
	did not provide their nationality	*N* = 1 (0.1%)	

**Nationalities of the ‘Latin West’ countries (nationalities included in the analysis)**		**Number of respondents – including those with multiple nationalities (% of final sample)**	**Multiple nationalities**
**1**	**Hungarian**	*N* = 441 (57.8%)	British (*N* = 1) Swedish (*N* = 1) Central European mix (*N* = 1)
**2**	**American**	*N* = 88 (11.5%)	British (*N* = 1) Spanish (*N* = 1)
**3**	**British**	*N* = 40 (5.2%)	American (*N* = 1) Australian (*N* = 2) Hungarian (*N* = 1) Norwegian (*N* = 1)
**4**	**Austrian**	*N* = 26 (3.4%)	French (*N* = 1)
	**Estonian**	*N* = 26 (3.4%)	-
**5**	**German**	*N* = 24 (3.1%)	-
**6**	**Australian**	*N* = 15 (2.0%)	British (*N* = 2)
**7**	**Canadian**	*N* = 14 (1.8%)	-
	**French**	*N* = 14 (1.8%)	Austrian (*N* = 1)
**8**	**Dutch**	*N* = 12 (1.6%)	Danish & Luxembourgish (*N* = 1)
	**Spanish**	*N* = 12 (1.6%)	American (*N* = 1)
**9**	**Brazilian**	*N* = 8 (1.0%)	-
	**Finnish**	*N* = 8 (1.0%)	-
	**Italian**	*N* = 8 (1.0%)	-
**10**	**Swedish**	*N* = 5 (0.7%)	Hungarian (*N* = 1)
**11**	**Norwegian**	*N* = 4 (0.5%)	British (*N* = 1)
	**Slovenian**	*N* = 4 (0.5%)	-
**12**	**Danish**	*N* = 3 (0.4%)	Dutch & Luxembourgish (*N* = 1)
	**Polish**	*N* = 3 (0.4%)	-
	**Portuguese**	*N* = 3 (0.4%)	-
	**Scottish**	*N* = 3 (0.4%)	-
**13**	**Croatian**	*N* = 2 (0.3%)	-
	**Irish**	*N* = 2 (0.3%)	-
**14**	**Belgian**	*N* = 1 (0.1%)	-
	**Central European mix**	*N* = 1 (0.1%)	Hungarian (*N* = 1) *No other clarification*
	**Czech**	*N* = 1 (0.1%)	-
	**Maltese**	*N* = 1 (0.1%)	-
	**Mexican**	*N* = 1 (0.1%)	-
	**Slovak**	*N* = 1 (0.1%)	-
	**Swiss**	*N* = 1 (0.1%)	-
	**Venezuelan**	*N* = 1 (0.1%)	-

The age range was 18–81 (median 41 ± 12.6 years). As common in similar studies^8,48,69,74,79,84,87,93,94^, the majority of respondents were women (90.2%) and owners of the animal species in question (94.8%). 32.0% of respondents reported that they own or had owned at least one brachycephalic dog.

### Questionnaire design

The Google Forms platform was used to build the questionnaire. The questionnaire was developed in English and tested with a small group of pilot participants to ensure it was easy to complete and would take no more than 15 min to fill out. A Hungarian version of the questionnaire, identical in content, was created by ZB and EK in order to take advantage of the established Hungarian follower base of the Family Dog Project’s Facebook page and group. The questionnaire was completed in either English or Hungarian.

The Google Forms platform did not store incomplete responses. If participants did not submit their answers at the end of the questionnaire, their data were not saved. Consequently, only responses from those who finished the questionnaire were recorded. The form only stored the submission timestamp; thus, no data is available regarding the completion time or dropout rate. The participants were aware that the completion time was approximately 15 min.

The study included questions on respondents’ demographics, personality, assessment of pre-existing attitudes towards brachycephalic dogs, knowledge of health problems, expectations of a hypothetical ideal dog, and favouring shorter muzzle length (assessed via photo ratings). The questionnaire was anonymous. All questions were required, except those related to the assessment of pre-existing attitudes towards brachycephalic dogs. For the English version of the questionnaire, see Supplementary Material I.

#### Respondents’ characteristics

##### Demography

Respondents were asked about their gender, age, nationality, and place of residence (classified according to the report of the Organisation for Economic Co-operation and Development^97^ to ensure international comparability), as well as whether they had ever lived with a dog and participated in its care.

We assessed respondents’ relationship with children by asking how many children they had, whether they want (more) children now or in the future, and whether they currently care for a child under the age of six at least once a week.

We recorded respondents’ highest level of education (using the International Standard Classification of Education (ISCED 2011)^98^ to ensure international comparability). Additionally, we asked whether they had received education beyond the basic level in health or natural sciences, and whether they had dog-related professional expertise (e.g. veterinarian, dog trainer, dog groomer).

##### Personality

To assess respondents’ personality, we used the 44-item Big Five Inventory^99^ and its Hungarian translation^100^ in the Hungarian version of the questionnaire.

Detailed proportions of the responses are presented in Supplementary Table 2.

##### Pre-existing attitudes towards brachycephalic dogs

Pre-existing attitudes were assessed indirectly to avoid biasing responses by explicitly mentioning brachycephalic dogs, following the approach used in previous research^74^. Respondents were asked to describe: (A) the appearance or breed of their own dog, (B) a dog breed or appearance they particularly liked, and (C) a dog breed or appearance they particularly disliked. These questions were optional. Question (A) was not answered by 5.4% of respondents, question (B) by 24.8%, and question (C) by 31.3%. In total, 0.9% of respondents did not answer any of the three questions. Based on their responses, participants were categorised as having a positive, negative, or neutral attitude towards brachycephalic dogs.

##### Knowledge of health problems

To assess how many health problems respondents associated with brachycephalism, we included a ‘health quiz’ in the questionnaire (available in Supplementary Material I). This quiz aimed to assess lay knowledge of breed-related disorders and was adapted without modification from a previous study^74^. Participants were asked to link seven health problems to three popular dog breeds. Each problem could be associated with one or more breeds. It was not required to associate every health problem with at least one breed, nor to link each breed to at least one problem in order to proceed with the questionnaire. Subsequently, we counted the number of health problems that respondents associated with the brachycephalic breed.

#### Expectations of a hypothetical ideal dog

We assessed respondents’ expectations of a hypothetical ideal dog by asking them about its traits (using items adapted from previous research^94,95^, its perceived role within the respondent’s social network^91–93^, and the aspects of dog ownership they find most rewarding (using statements from previous research^93^, supplemented with additional items derived from qualitative data collected from brachycephalic dog owners^71^. All items are provided in Supplementary Material I.

#### Preference for shorter muzzle length (visual preference task)

To exclude other factors that might influence respondents’ preference—such as coat colour, coat length, or ear shape—we used manipulated images of the same individual dogs, ensuring that muzzle length was the only differing feature. Profile images of 14 mesocephalic mongrels were used to minimise the influence of breed-related preconceptions and to produce visually similar photo sets. Each dog was presented in four versions with digitally modified muzzle lengths: much longer (+ 15%), slightly longer (+ 5%), slightly shorter (−5%), and much shorter (−15%) than the original (see examples in Fig. 1; full set in Supplementary Table 1). The four versions were presented simultaneously, and respondents were asked to indicate which one they liked most (A/B/C/D). The order of muzzle lengths within each dog’s image set was semi-randomised, and the order of dog presentations was fully randomised across participants.

### Photo manipulation

Muzzle length was manipulated by morphing the original profile photographs. To avoid creating unnatural appearances—such as purebred dogs with atypical muzzle proportions—we selected only mongrel dogs without clearly recognisable breed characteristics. Furthermore, to standardise the extent of manipulation across all images, and manipulating muzzle length to a higher degree than 15% shortening or elongating resulted in unnatural aesthetics, we chose only mesocephalic mongrels for this purpose. As a result, our (morphed) photo stimuli did not include extremely short-headed or long-headed dogs, although preferences for shorter muzzles could still be meaningfully investigated.

First, as a reference for morphing, we prepared profile images of eight dolichocephalic dogs and eight brachycephalic dogs. They were taken from the Internet. Fifty landmarks were delineated on each face profile by using tpsdig2 software (version 2.31^101^). Then, we superimposed them and created the average dolichocephalic dog and brachycephalic dog faces by using Webmorph^102^. Based on the landmark delineation of these average faces, we transformed the profile shape of fourteen mesocephalic mongrel dogs. The photographs of the dogs were taken from other studies^3,103,104^ and included only mongrel dogs without recognisable breed types. The transformation was made either + 15%, + 5%, −5%, or −15% toward the average dolichocephalic profile, resulting in no extremely short or long muzzle in the stimuli (Fig. 1). There should not be huge variations in muzzle length among the original dogs because they were all from mesocephalic dogs. However, there were still some variations, so it is possible that the same transformation (e.g., + 5%) resulted in a different outcome of muzzle length depending on the original muzzle length of the fourteen dogs.

Although we took actions to avoid unnatural manipulations, we wanted to be sure that we did not measure the effect of unnaturalness in the analysis. Therefore, in an independent sample of 24 respondents, we assessed how people perceived the unnaturalness of the photos. The photos were presented one by one in a semi-random order. For each photo, respondents were asked, ‘Does this dog look unnatural?’ They had to rate this question on a Likert scale, where 1 meant ‘not at all’ and 5 meant ‘very much’. The proportion of their responses was used to calculate an ‘unnaturalness score’ for each photo. Then, we calculated the Relative Unnaturalness Rank of each muzzle length of each photo. The least unnatural received a score of 0, while the scores for the other three were calculated relative to the least unnatural one. These ranks are presented in Supplementary Table 1.

### Statistical analysis

We used R 4.2.2 software^105^ for statistical analysis.

#### Data cleaning

Free-text responses assessing participants’ pre-existing attitudes towards brachycephalic dogs were coded by ZB. Attitudes were categorised as follows:

- *Positive*: participants who live or had lived with a brachycephalic dog and/or explicitly expressed a liking for brachycephalism, brachycephalic breeds, or related features in dog appearance.
- *Negative*: participants who explicitly expressed a dislike for brachycephalism, brachycephalic breeds, or related features in dog appearance.
- *Neutral*: participants who did not express either a liking or a dislike for brachycephalism, brachycephalic breeds, or related features.

Variables with low variance were excluded from analysis. This included: [1] gender (90.2% were women); [2] number of children born (64.2% of respondents had no children); [3] future children (66.2% of respondents do not want any children); [4] currently taking care of a child under the age of 6 at least once a week (87.5% of respondents do not); and [5] dog ownership (94.8% of respondents own/owned a dog). From questions regarding the traits of the ideal dog: [6] shows affection towards the owner (96.7% of the respondents agreed); [7] fully housetrained (91.2% of the respondents agreed); [8] physically healthy (92.5% of the respondents agreed); and [9] lives until he/she is at least 10 years old (88.9% of the respondents agreed). From questions regarding the role of the ideal dog: [10] friend (91.0% of the respondents agreed); and [11] family member (94.1% of the respondents agreed). Regarding what is rewarding in dog keeping: [12] stroking, contact (93.1% of the respondents agreed); [13] providing company (89.5% of the respondents agreed); and [14] unconditional love (88.1% of the respondents agreed). Detailed proportions of the responses are presented in Supplementary Tables 2 and 3.

In order to reduce dimensionality among the questions regarding the *Traits of the ideal dog* and *Rewarding in dog keeping*, that is, summarise the related variables into summary indexes, we used Principal Component Analysis (PCA, ‘principal’ functions of ‘psych’ package^106^. First, we checked whether the datasets from the two language versions (Hungarian and English) of the questionnaire could be merged by running PCA separately on the Hungarian and English answers to ensure that we have the same component structure in both languages. Then, after merging the answers, we performed a parallel analysis to determine the number of components to extract (‘fa. parallel’ function of ‘psych’ package^106^, then we utilized a PCA on the items of the questions. After calculating the component scores, we checked the internal consistency of the scores with Cronbach’s alpha (‘cronbach.alpha’ function of ‘ltm’ package^107^.

#### Factors influencing the preference for shorter muzzle length in dog images

Firstly, we investigated whether there was an association between the Relative Unnaturalness Rank and the frequency of muzzle length choice in the 14 photo stimuli. Since the data were not normally distributed and the sample size was small, we used Kendall’s rank correlation (‘cor.test’ function of ‘stats’ package^105^. We excluded five problematic photo quadruplets, for which the Relative Unnaturalness Rank was skewed, from further analysis (for details, see Supplementary Table 1).

*Preference for shorter muzzle length* was treated as an ordinal variable; therefore, ordinal regression was conducted. We used Cumulative Link Mixed Models (‘clmm’ function of ‘ordinal’ package^108^ to examine the association between the *Preference for shorter muzzle length* and respondents’ characteristics, pre-existing attitudes towards brachycephalic dogs, knowledge of health problems, and expectations of a hypothetical ideal dog. Respondent ID and the photo ID were included as random effects.

We built four models:

Model 1: included all respondents from the ‘Latin West’ countries.

Model 2: included only respondents with pre-existing positive attitude towards brachycephalic dogs.

Model 3: included only respondents without pre-existing attitude (neutral attitude).

Model 4: included only respondents with pre-existing negative attitude.

Bottom-up, AIC-based model selection procedures were used to find the most parsimonious models (‘anova’ function of ‘stats’ package^105^. Variables were retained if they significantly improved model fit based on a likelihood ratio test and yielded at least a two-point improvement in AIC.

Odds ratios (OR) and their 95% confidence intervals (CI) were calculated from the model estimates. Specifically, odds ratios were computed using the formula OR = exp(β), where β is the model estimate. The 95% confidence intervals were calculated as CI = [exp(β − 1.96 × SE), exp(β + 1.96 × SE)], where SE is the standard error of the estimate.

Variance inflation factor (VIF; ‘vif’ function of the ‘car’ package^109^ was used to assess the possibility of multicollinearity among the independent variables.

## Results

33.0% of respondents were categorised as having a neutral attitude towards brachycephalic dogs. 29.0% of respondents were categorised as having a positive attitude, 93.7% of them lived with a brachycephalic dog currently or previously. 38.0% of respondents were categorised as having a negative attitude. 12.8% of respondents with a negative attitude lived with a brachycephalic dog currently or previously but expressed a stronger negative attitude towards brachycephalic dogs as a dog type they dislike. Additional characteristics of the respondents in the final sample (*N* = 763) are summarised in Supplementary Table 2, while Supplementary Table 3 presents the proportions of responses regarding their expectations of a hypothetical ideal dog.

Principal component analysis (PCA) revealed a single component encompassing three items related to what participants find rewarding in dog keeping: [1] admiration the dog’s appearance (loading = 0.839); [2] taking pictures of the dog (loading = 0.883); and [3] receiving acknowledgment from others that the dog is beautiful/cute (loading = 0.846). We named the component *Appreciation of aesthetics* (Cronbach’s alpha = 0.769; SS loadings = 2.199; proportion of variance = 0.733).

Respondents generally preferred shorter muzzle lengths. They chose the shorter lengths in 66.7% of the cases, indicating a stronger preference for shorter muzzle lengths and a lesser preference for longer lengths. The shortest muzzles (shortened by 15%) were chosen in 29.0% of the cases. The slightly shortened muzzles (shortened by 5%) were chosen most often, in 37.7% of the cases. The slightly elongated muzzles (elongated by 5%) were chosen in 23.4% of the cases. The longest muzzles (elongated by 15%) were chosen the least frequently, in only 9.9% of the cases.

### Factors influencing the preference for shorter muzzle length among respondents from ‘latin west’ countries

**Fig. 2:**
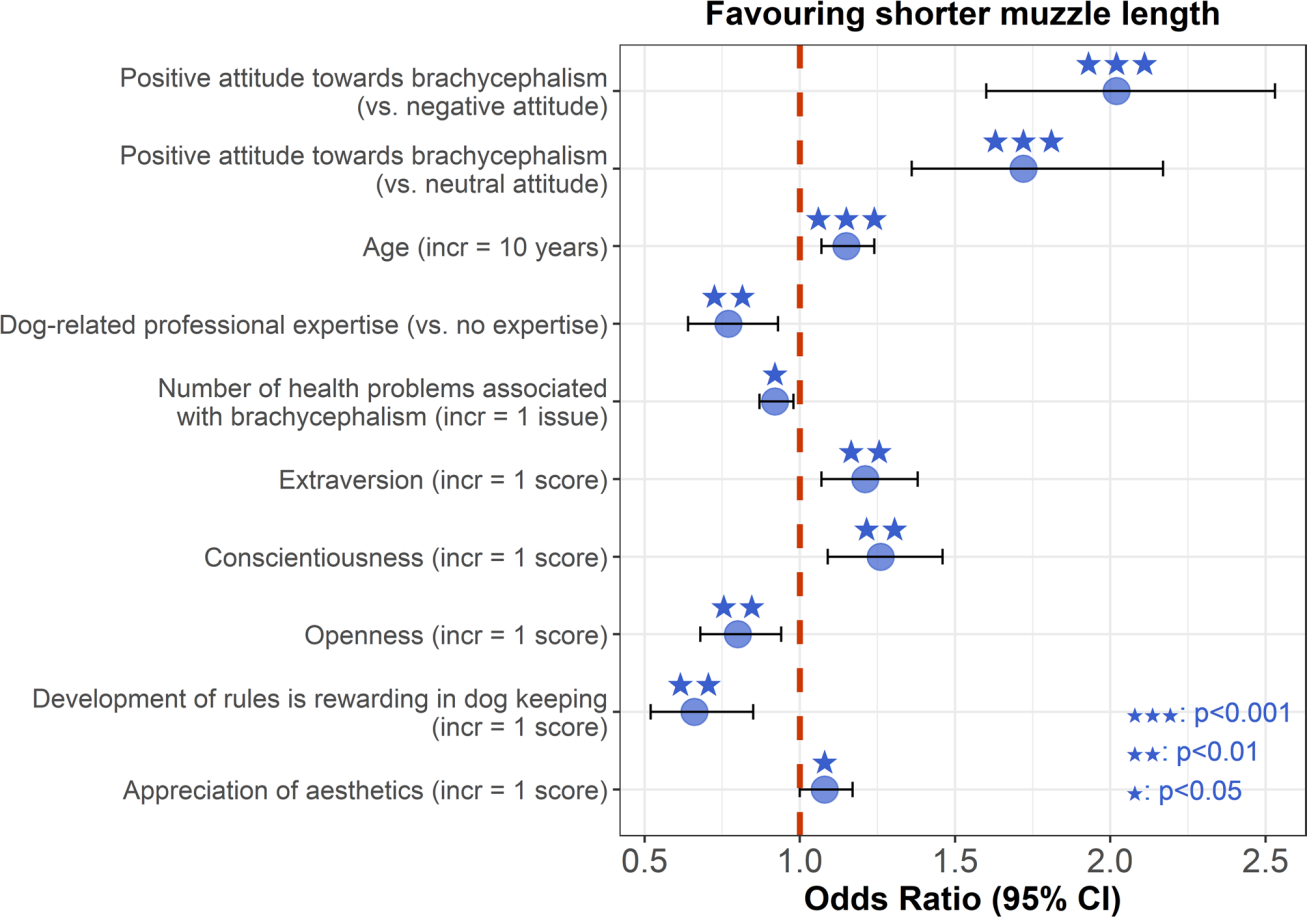
Odds ratios for factors significantly influencing the preference for shorter muzzle length.

We identified nine factors that influenced the preference for shorter muzzle length among respondents from ‘Latin West’ countries (Fig. 2). The variance inflation factor scores (VIF) indicated no multicollinearity among the independent variables. The nine factors were: [1] pre-existing attitudes towards brachycephalic dogs (VIF = 1.03); [2] age (VIF = 1.06); [3] dog-related professional expertise (VIF = 1.05); [4] number of health problems associated with brachycephalism (VIF = 1.03); [5] extraversion (VIF = 1.09); [6] conscientiousness (VIF = 1.04); [7] openness (VIF = 1.06); [8] development of rules and control is rewarding in dog keeping (VIF = 1.03); and [9] appreciation of aesthetics (VIF = 1.05). The variance explained by the respondents’ ID was 1.18 ± 1.08 and by the pictures’ ID was 0.22 ± 0.46. Hungarian respondents did not differ significantly from non-Hungarians (*p* = 0.148).

**Pre-existing attitudes towards brachycephalic dogs.** Respondents with a positive attitude towards brachycephalic dogs were more likely to prefer shorter muzzle lengths compared to those with a negative attitude or a neutral attitude (negative: ß ± SE: 0.70 ± 0.12; Z = 6.00; OR = 2.02 [1.60–2.53]; *p* < 0.001; neutral: ß ± SE: 0.54 ± 0.12; Z = 4.53; OR = 1.72 [1.36–2.17]; *p* < 0.001).

**Age.** Older respondents were more likely to prefer shorter muzzle lengths (*p* < 0.001).

**Dog-related professional expertise.** Respondents with a dog-related professional expertise were less likely to prefer shorter muzzle lengths compared to those without expertise (*p* = 0.008).

**Number of health problems associated with brachycephalism.** Respondents who associated more health problems with brachycephalism were less likely to prefer shorter muzzle lengths (*p* = 0.012).

**Extraversion.** Respondents scoring higher on extraversion were more likely to prefer shorter muzzle lengths (*p* = 0.003).

**Conscientiousness.** Respondents scoring higher on conscientiousness were more likely to prefer shorter muzzle lengths (*p* = 0.002).

**Openness.** Respondents scoring higher on openness were less likely to prefer shorter muzzle lengths (*p* = 0.008).

**Development of rules is rewarding in dog keeping.** Individuals who found development of rules rewarding in dog keeping were less likely to prefer shorter muzzle lengths (*p* = 0.001).

**Appreciation of aesthetics.** Respondents who appreciated aesthetics more were more likely to prefer shorter muzzle lengths (*p* = 0.044).

Detailed outcomes of the final models are presented in Table 2.

### Factors influencing the preference for shorter muzzle length, depending on pre-existing attitudes towards brachycephalic dogs

**Fig. 3:**
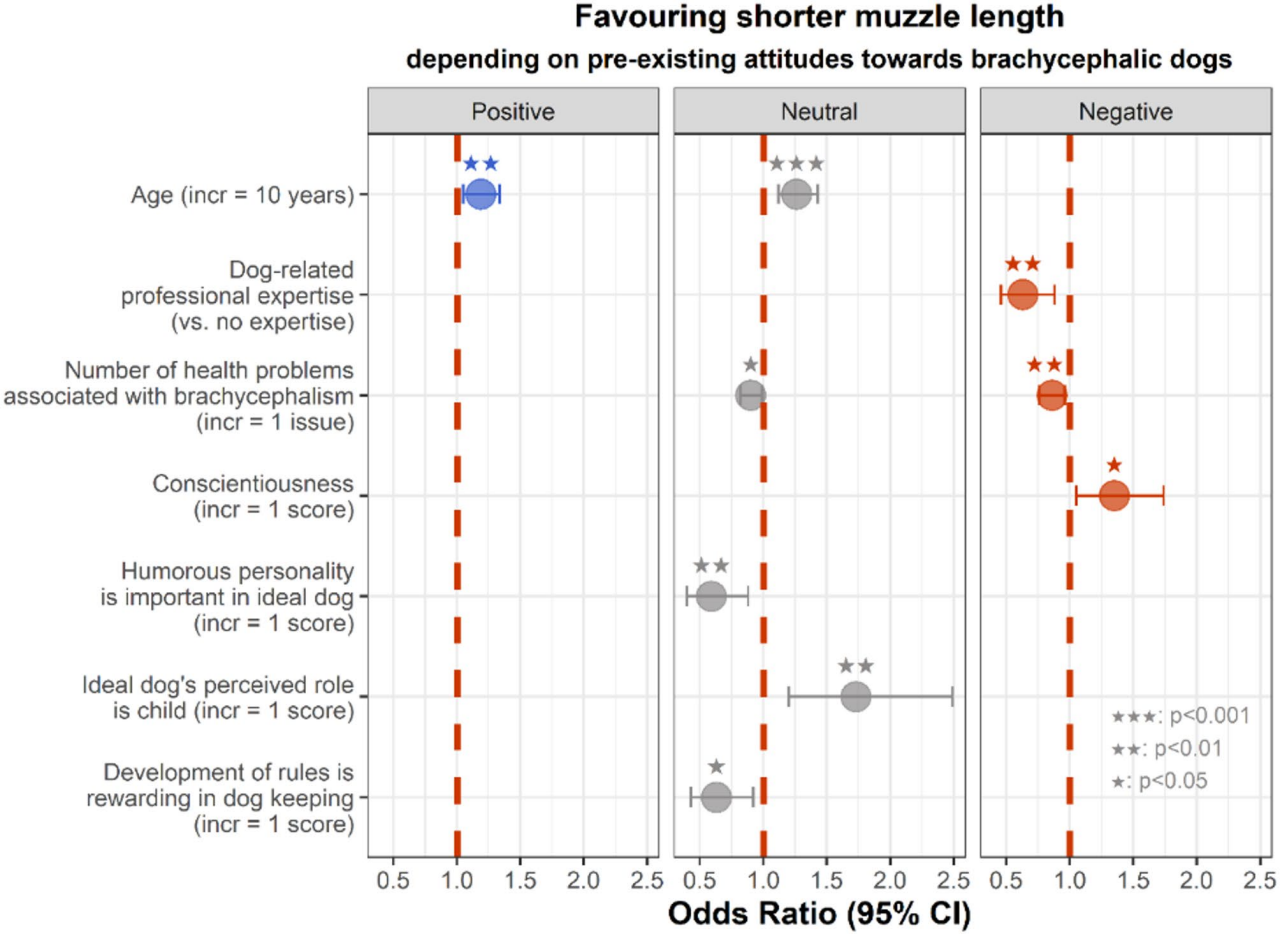
Odds ratios for factors significantly influencing the preference for shorter muzzle length, depending on pre-existing attitudes towards brachycephalic dogs.

We identified only one factor that influenced the preference for shorter muzzle length among respondents who held a pre-existing positive attitude towards brachycephalic dogs (Fig. 3). The variance explained by the respondents’ ID was 1.00 ± 1.00 and by the pictures’ ID was 0.11 ± 0.33.

We identified five factors that influenced the preference for shorter muzzle length among respondents who did not hold any pre-existing attitude towards brachycephalic dogs (neutral attitude) (Fig. 3). The variance inflation factor scores (VIF) indicated no multicollinearity among the independent variables. The five factors were: [1] age (VIF = 1.02); [2] number of health problems associated with brachycephalism (VIF = 1.02); [3] humorous personality is an important trait of the ideal dog (VIF = 1.03); [4] child is the role of the ideal dog (VIF = 1.03); and [5] development of rules is rewarding in dog keeping (VIF = 1.03). The variance explained by the respondents’ ID was 1.06 ± 1.03 and by the pictures’ ID was 0.58 ± 0.76.

We identified three factors that influenced the preference for shorter muzzle length among respondents who held a pre-existing negative attitude towards brachycephalic dogs (Fig. 3). The variance inflation factor scores (VIF) indicated no multicollinearity among the independent variables. The three factors were: [1] dog-related professional expertise (VIF = 1.04); [2] number of health problems associated with brachycephalism (VIF = 1.04); and [3] conscientiousness (VIF = 1.00). The variance explained by the respondents’ ID was 1.52 ± 1.23 and by the pictures’ ID was 0.43 ± 0.66.

**Age.** Among respondents with a pre-existing positive or neutral attitude, older individuals were more likely to prefer shorter muzzle lengths (positive: *p* = 0.008; neutral: *p* < 0.001).

**Dog-related professional expertise.** Among respondents with a pre-existing negative attitude, individuals with a dog-related professional expertise were less likely to prefer shorter muzzle lengths compared to those without an expertise (*p* = 0.007).

**Number of health problems associated with brachycephalism.** Among respondents with a pre-existing negative or neutral attitude, individuals who associated more health problems with brachycephalism were less likely to prefer shorter muzzle lengths (negative: *p* = 0.007; neutral: *p* = 0.036).

**Conscientiousness.** Among respondents with a pre-existing negative attitude, individuals scoring higher on conscientiousness were more likely to prefer shorter muzzle lengths (*p* = 0.022).

**Humorous personality is an important trait of the ideal dog.** Among respondents without a pre-existing attitude (neutral attitude), preference for shorter muzzle lengths was negatively associated with the importance attributed to a humorous personality in the ideal dog (*p* = 0.009).

**Child is the role of the ideal dog.** Among respondents without a pre-existing attitude (neutral attitude), individuals who were more inclined to perceive the ideal dog as a child were more likely to prefer shorter muzzle lengths (*p* = 0.003).

**Development of rules is rewarding in dog keeping.** Among respondents without a pre-existing attitude (neutral attitude), individuals who found development of rules rewarding in dog keeping were less likely to prefer shorter muzzle lengths (*p* = 0.018).

Detailed outcomes of the final models are presented in Table 1.

**Table 2:** Detailed outcomes of the four models. All significant results are reported in detail. Non-significant p-values originate from the final steps of model selection, where tested variables did not significantly improve model fit and were therefore excluded from the final models.

Models	Model 1: All respondents from the ‘Latin West’ countries	Pre-existing attitudes towards brachycephalic dogs		
		Model 2: Only respondents with positive attitude	Model 3: Only respondents with neutral attitude	Model 4: Only respondents with negative attitude
**Age**	ß ± SE: 0.14 ± 0.04; Z = 3.69; OR = 1.15 [1.07–1.24]; incr = 10 years; *p* < 0.001 ***	ß ± SE: 0.17 ± 0.06; Z = 2.64; OR = 1.19 [1.05–1.34]; incr = 10 years; *p* = 0.008 **	ß ± SE: 0.23 ± 0.06; Z = 3.73; OR = 1.26 [1.12–1.43]; incr = 10 years; *p* < 0.001 ***	*p* = 0.267
**Dog-related professional expertise**	ß ± SE: −0.26 ± 0.10; Z = −2.67; OR = 0.77 [0.64–0.93]; *p* = 0.008 **	*p* = 0.264	*p* = 0.865	ß ± SE: −0.46 ± 0.17; Z = −2.72; OR = 0.63 [0.46–0.88]; *p* = 0.007 **
**Highest level of education**	*p* = 0.190	*p* = 0.499	*p* = 0.282	*p* = 0.393
**Advanced level of education in health or natural science**	*p* = 0.639	*p* = 0.146	*p* = 0.839	*p* = 0.387
**Number of health problems associated with brachycephalism**	ß ± SE: −0.08 ± 0.03; Z = −2.52; OR = 0.92 [0.87–0.98]; *p* = 0.012 *	*p* = 0.603	ß ± SE: −0.10 ± 0.05; Z = −2.10; OR = 0.90 [0.82–0.99]; *p* = 0.036 *	ß ± SE: −0.16 ± 0.06; Z = −2.70; OR = 0.86 [0.76–0.96]; *p* = 0.007 **
**Personality**				
**Extraversion**	ß ± SE: 0.19 ± 0.06; Z = 2.99; OR = 1.21 [1.07–1.38]; *p* = 0.003 **	*p* = 0.219	*p* = 0.066.	*p* = 0.198
**Agreeableness**	*p* = 0.481	*p* = 0.170	*p* = 0.131	*p* = 0.897
**Conscientiousness**	ß ± SE: 0.23 ± 0.07; Z = 3.15; OR = 1.26 [1.09–1.46]; *p* = 0.002 **	*p* = 0.113	*p* = 0.094.	ß ± SE: 0.30 ± 0.13; Z = 2.30; OR = 1.35 [1.05–1.74]; *p* = 0.022 *
**Neuroticism**	*p* = 0.162	*p* = 0.762	*p* = 0.397	*p* = 0.361
**Openness**	ß ± SE: −0.22 ± 0.08; Z = −2.65; OR = 0.80 [0.68–0.94]; *p* = 0.008 **	*p* = 0.875	*p* = 0.497	*p* = 0.066
**Traits of the hypothetical ideal dog**				
**Safe with children**	*p* = 0.158	*p* = 0.320	*p* = 0.263	*p* = 0.308
**Beautiful**	*p* = 0.102	*p* = 0.273	*p* = 0.997	*p* = 0.495
**Low exercise requirements**	*p* = 0.983	*p* = 0.570	*p* = 0.413	*p* = 0.287
**Humorous personality**	*p* = 0.610	*p* = 0.147	ß ± SE: −0.52 ± 0.20; Z = −2.61; OR = 0.59 [0.40–0.88]; *p* = 0.009 **	*p* = 0.927
**Role of the hypothetical ideal dog**				
**Fellow worker**	*p* = 0.335	*p* = 0.306	*p* = 0.686	*p* = 0.532
**Pet**	*p* = 0.486	*p* = 0.563	*p* = 0.548	*p* = 0.376
**Child**	*p* = 0.103	*p* = 0.601	ß ± SE: 0.55 ± 0.19; Z = 2.92; OR = 1.73 [1.20–2.49]; *p* = 0.003 **	*p* = 0.747
**A dog is more important to me than any other person**	*p* = 0.717	*p* = 0.883	*p* = 0.949	*p* = 0.513
**Rewarding in dog keeping**				
**Security**,** watchdog**	*p* = 0.606	*p* = 0.193	*p* = 0.973	*p* = 0.759
**Teaching**,** training**,** sports**	*p* = 0.361	*p* = 0.248	*p* = 0.772	*p* = 0.689
**Taking care of someone**	*p* = 0.334	*p* = 0.159	*p* = 0.327	*p* = 0.927
**Development of rules**	ß ± SE: −0.41 ± 0.13; Z = −3.22; OR = 0.66 [0.52–0.85]; *p* = 0.001 **	*p* = 0.972	ß ± SE: −0.46 ± 0.20; Z = −2.36; OR = 0.63 [0.43–0.92]; *p* = 0.018 *	*p* = 0.329
**Contact with other people (e.g. other dog owners)**	*p* = 0.499	*p* = 0.867	*p* = 0.278	*p* = 0.532
**Dog walking**	*p* = 0.176	*p* = 0.489	*p* = 0.782	*p* = 0.139
**Entertainment/play**	*p* = 0.303	*p* = 0.417	*p* = 0.069.	*p* = 0.069
**Appreciation of aesthetics**	ß ± SE: 0.08 ± 0.04; Z = 2.02; OR = 1.08 [1.00–1.17]; *p* = 0.044 *	*p* = 0.243	*p* = 0.072.	*p* = 0.999

## Discussion

We examined how muzzle length, as the only varying feature, influenced visual preference for dogs, using digitally morphed images of the same individuals to eliminate the influence of other physical or breed-related characteristics. Our broader aim was to explore which human-related factors influence the preference for shorter muzzles. Such preferences may be among the drivers behind the trend towards increasingly shorter average muzzle lengths in dog populations across the Western world^4–9^, ultimately contributing to the ‘brachycephalic crisis’^19^. Brachycephalism is associated with well-documented predisposition to a wide range of serious health problems, including brachycephalic obstructive airway syndrome (BOAS)^20–23^, gastrointestinal disorders^26,27^, thermoregulatory difficulties^25,28^, exercise intolerance^25^, sleep apnoea^24,25^, ophthalmological disorders^32–34^, dental abnormalities^19^, dermatological conditions^35,36^, brain disorders^19,37^, and reproductive issues^19,41,42^. In addition, many brachycephalic breeds are prone to vertebral and spinal malformations^38–40^. Due to the cumulative burden of these conditions, brachycephalic dogs also show increased risk of early mortality^19,29–31^. However, public awareness of brachycephalic health issues remains limited or is actively disregarded by brachycephalic dog owners and enthusiasts^70,74,81–85^. By manipulating only muzzle length in images of dogs presented in our visual preference task, keeping all other morphological traits constant, we aimed to determine whether the preference for shortened muzzles persists independently. Furthermore, by investigating demographic variables, personality traits, pre-existing attitudes towards brachycephalic dog breeds, and awareness of health issues, we aimed to identify the psychological and social factors that sustain consumer demand for dogs with extreme conformations.

We have found that respondents generally preferred shorter muzzle lengths, despite the smaller number of respondents with a pre-existing positive attitude towards brachycephalic dogs (*N* = 221) compared to those with negative (*N* = 290) and neutral (*N* = 252) attitudes in our sample. This aligns with previous findings in rabbits, where shorter-headed individuals were globally preferred over longer-headed ones^80^.

Furthermore, our results showed that respondents who held a pre-existing positive attitude towards brachycephalic dogs^74^ were significantly more likely to prefer shorter muzzle lengths compared to those with neutral or negative attitudes. A similar pattern was observed in cats, where owners’ preferences aligned with the skull shape of their own cat^79^. The present study is the first to empirically demonstrate, using controlled stimuli, that the attractiveness of brachycephalic dogs is partly driven by humans’ preference for shorter muzzles in dogs.

We also identified several other factors influencing the preference for shorter muzzles. These include demographic factors such as age and dog-related professional expertise, the number of health problems respondents associated with brachycephalism, personality traits such as extraversion, conscientiousness, and openness, and expectations related to the ideal dog. Specifically, preferences were influenced by traits like the perceived role of the dog as a child, the importance of a humorous personality, and the rewarding nature of rule development and appreciation of aesthetics in dog keeping.

### Associations with demographic factors

We initially expected a negative association between age and preference for shorter muzzle length, as previous research suggests that younger individuals tend to have more positive attitudes towards brachycephalic dogs^74^, potentially due to their greater sensitivity to the baby schema effect^75^. Contrary to our earlier findings^74^, but in line with other studies reporting that brachycephalic dog breeds are predominantly owned by older adults^8^, our results showed that older respondents were more likely to prefer shorter muzzle lengths. Inconsistencies in age-related associations with the baby schema effect have also been noted in previous research^75^. One possible explanation is that older individuals may perceive shorter-headed dogs as less active and, therefore, more manageable^71^. Alternatively, this pattern may reflect an aversion to longer-headed dogs, which could be perceived as more active or aggressive—possibly due to associations with hunting or working dog breeds.

Although previous research has found that women and parents tend to show more positive attitudes towards brachycephalic dogs^74^, which may also stem from a similar sensitivity to infant-like traits^75–78^, the present study could not investigate this factor in relation to preference for shorter muzzle lengths, as the majority of the sample consisted of childless women.

### Associations with professional expertise, education, and awareness of health issues

Previous research has shown that individuals with animal-related professional expertise are more likely to have a negative attitude towards brachycephalic animals^74,79,80^, likely due to their deeper understanding and awareness of the health and welfare issues associated with extreme brachycephaly. In the present study, we observed that respondents with dog-related professional expertise were less likely to prefer shorter muzzles. This may be because professionals tend to favour the longer-headed, ‘typical dog’ appearance and are less influenced by the baby schema effect in dogs. In contrast, individuals without professional expertise may be more susceptible to the portrayals of dogs in popular culture and media, where shorter-headed dogs are often depicted as ‘cute’ and appealing^110^, with the health risks associated with extreme brachycephaly being overlooked. These individuals may place greater emphasis on the aesthetic qualities of shorter-headed dogs rather than considering the potential health and ethical concerns associated with extreme brachycephalic breeds^69^.

Brachycephalic dog owners often normalise and accept the health problems as inherent traits of these dogs, meaning that awareness of the associated health risks does not necessarily deter them from choosing such dogs^8,73,74,81–85^. In line with this, our findings revealed that when assessing preferences for shorter muzzle lengths, individuals who associated more health problems with brachycephalism were less likely to prefer shorter muzzles—but this did not hold true for those with pre-existing positive attitudes towards brachycephalic dogs. Among this group, the number of health issues associated with brachycephalism had no influence on their preferences, even though they were found to attribute a greater number of health issues to brachycephalism^74^. Although we did not include extremely short muzzles as stimuli, the number of health issues attributed to brachycephaly still influenced preferences in participants with neutral or negative attitudes. These findings support the notion that brachycephalic dog enthusiasts’ awareness of health problems does not affect their attitudes towards increasingly shortened head shapes. Paradoxically, however, approximately 90% of respondents agreed that the ideal dog is physically healthy and lives at least 10 years, suggesting that the concept of ‘healthy’ may hold a different meaning for brachycephalic dog enthusiasts.

Previously, higher levels of education have been found to be associated with more negative attitudes towards brachycephalic animals^74,79,80^, possibly because higher education often fosters critical thinking and a more analytical approach to information. We also hypothesised that having an educational background in health-related or natural science fields might influence people’s preferences for muzzle length, as such educational backgrounds may support a more critical engagement with information regarding the health and welfare concerns of brachycephalic dogs, which is increasingly disseminated via social media platforms as a result of awareness campaigns^88–90^. However, we found no association between education level, or having advanced education in health or natural sciences, and respondents’ preferences for shorter head shapes.

### Associations with human personality traits

Our results support the notion that personality traits influence individuals’ attitudes towards dog breeds and their aesthetic preferences. Specifically, respondents scoring higher on conscientiousness and extraversion were more likely to prefer shorter muzzle lengths, whereas those with higher openness scores were less inclined to do so. The widespread popularity of brachycephalic dog breeds in the Western world^4–8^ can be interpreted as a sociocultural norm favouring shorter-headed dogs. In this context, our findings align with previous research showing that conscientious and extraverted individuals tend to conform to such norms, while individuals high in openness are more inclined to resist them^86^.

Yet, the underlying mechanisms may differ across personality traits^86^. For conscientious individuals, adhering to sociocultural norms is typically aligned with rational decision-making, which may explain their tendency to conform^86,111^. In line with this, previous research found that individuals with more negative attitudes towards brachycephalic dogs tend to score lower on conscientiousness^74^, possibly because they are more inclined to intentionally deviate from social norms. As conscientious individuals are more inclined towards control and planning, they may perceive longer-headed dogs as more challenging to manage and less cooperative. This could explain why they are less likely to prefer these head shapes, as they may associate them with greater difficulty in handling and living with them.

Extroverted individuals often seek positive social attention and may align with sociocultural norms as a way of fulfilling this desire^86^. Because extroverts are more attuned to the social environment and seek validation and positive support from others^111^, they may be more inclined to prefer dogs with an ‘attention-grabbing’ appearance, which promotes social interactions and recognition from others^72^. However, no association was found between preference for muzzle length and the enjoyment of social contact with other people. Their strong inclination for social engagement^111^ likely extends to their choice of companion animals. They may perceive shorter-muzzled dogs as more approachable and friendly, likely because they resemble infants due to baby schema traits^10,11,15^. Research suggests that extraverted individuals are less likely to engage in intellectual investment^112^, which may imply that they are less aware of the welfare issues associated with shorter muzzle lengths in dogs.

In contrast, openness has been positively linked to general knowledge, suggesting that individuals high in openness are more likely to invest in and seek out information^112^. Open individuals tend to be curious and intellectually engaged. Consistent with this, they are also more interested in discussing natural processes, including biological phenomena^111^. Openness may also predispose individuals to form alternative viewpoints and engage in independent thinking^86^. As a result, they may be more likely to prefer a more natural, ‘typical dog’ appearance and less influenced by the baby schema effect in dogs.

The previously observed association between agreeableness and attitudes towards brachycephalic dogs^74^ was not found in preferences for muzzle lengths.

### Associations with expectations regarding the ideal dog and the rewarding aspects of dog ownership

Approximately 90% of respondents agreed that the ideal dog is affectionate towards its owner; its perceived role is friend and family member; the rewarding aspects of dog ownership are the opportunity to stroke the dog, being in contact with it, and receive companionship and unconditional love. Thus, these factors are unlikely to be associated with a preference of shorter muzzle length in dogs.

We also found interesting associations between respondents’ expectations of a hypothetical ideal dog, the aspects of dog ownership they consider most rewarding, and their preference for shorter muzzle lengths in dogs. When asked in a previous study why they would recommend their brachycephalic breed to potential dog owners, current owners often emphasized the breed’s child-friendliness, ‘lazy’ temperament and ‘humorous’ personality^71^. However, in the present study, neither the importance placed on safety with children nor low exercise needs was associated with respondents’ preference for shorter muzzle lengths, suggesting that these traits may not be linked to morphology-related preconceptions in dogs. The importance of a humorous personality showed an association with shorter muzzle length preference only among respondents without pre-existing attitudes towards brachycephalic dogs—yet this association was negative. That is, individuals who valued a humorous personality more in an ideal dog were less likely to prefer shorter-headed dogs. This suggests that perceptions of humorous personality are likely tied to brachycephalic breed-related stereotypes rather than to the dogs’ morphological features.

We also observed only among respondents without pre-existing attitudes towards brachycephalic dogs, that individuals who were more inclined to perceive the ideal dog as a child were more likely to prefer shorter muzzle lengths. In the absence of established preference or aversion of brachycephalic dog breeds, people who see dogs as their children prefer shorter head shapes in dogs. This supports the theory that dogs may transform into child substitutes in modern Western societies^17^and humans create a selection pressure on dogs towards shorter and shorter muzzle lengths, and consequently a more infant-like facial appearance^10,11,15^. The decreased number of children requiring care has potentially created a new ecological niche for dogs^17^, what dogs with increasingly shorter heads are now starting to fill.

According to John Archer’s theory, dogs can be considered social parasites that manipulate human behaviour to enhance their own fitness^60^. Given the popularity of small-sized brachycephalic dogs^4–8^, these breeds can be considered evolutionarily successful. They are likely to manipulate human parental caregiving behaviours by exploiting the baby schema—an infantile set of traits known to trigger nurturing responses in humans^10,12–14^. Therefore, shorter-headed dogs may act as child substitutes, fulfilling human needs for social connection and parental caregiving behaviours^17,60^, while demanding significantly less time and energy investment than children. However, no significant association was found between preference for muzzle length and the extent to which respondents found caregiving-related aspects of dog ownership rewarding. This suggests that the preference for a shorter muzzle is not motivated by a desire to care for others, at least not consciously. Whether the social parasite theory is applicable to the popularity of small-sized brachycephalic breeds requires further investigation.

The increasingly exaggerated shorter muzzles of brachycephalic breeds have led to a range of serious conformation-related disorders^19–42^. These conditions often require increased caregiving efforts from owners, which may, in turn, strengthen the emotional bond between them and their dogs^59,60,72^. This is supported by findings indicating that owners of small brachycephalic dogs report particularly strong emotional bonds to their pets^59,84^. Nevertheless, the high level of maintenance required to manage these health problems is also a commonly cited reason why many brachycephalic dog owners would advise against these breeds^71^.

Regarding the rewarding aspects of dog ownership, we found that individuals who valued the development of rules as a rewarding component of dog keeping were less likely to prefer shorter muzzle lengths. These individuals may perceive longer-muzzled dogs as more trainable and responsive to rule-following, possibly due to their association with traditional working or hunting breeds. In contrast, brachycephalic breeds are often perceived as more ‘stubborn’ or less obedient^71^, which may be less attractive to those who derive satisfaction from structured training and the establishment of rules. However, no association was found between preference for muzzle length and enjoyment of teaching, training, or dog sports.

Previous studies have reported that appearance plays a more significant role in the decision to acquire a brachycephalic breed than in the selection of non-brachycephalic breeds^59,69^, and that owners of extremely brachycephalic dogs are more likely to share videos of their pets on social media^73^. In line with these reports, the present study also revealed a positive association between the aesthetics appreciation PCA score and preference for shorter muzzle length. Individuals who admire the dog’s appearance, enjoy taking photos of the dog, and appreciate receiving acknowledgment from others that the dog is beautiful/cute were more likely to prefer shorter muzzles. This further supports the notion that extrinsically motivated individuals are drawn to shorter-muzzled dogs. In extreme cases, such preferences may extend to breeds with exaggerated brachycephalic features, which could function as status symbols^72^.

### Limitations

One of the main limitations of our study was the lack of extremely short muzzles in the stimuli, although preferences for shorter muzzles could still be meaningfully investigated. The reason for this was that we tried to avoid violating people’s expectations of breeds’ head shape by using purebred dogs with modified muzzle lengths, which could be perceived as unnatural. Consequently, we chose photographs of mongrel dogs without recognisable breed types. Additionally, manipulating muzzle length to a higher degree than 15% shortening or elongating resulted in unnatural aesthetics. Since we wanted to create similar muzzle lengths for each dog, we chose only mesocephalic mongrels for this purpose. Still, we found evidence that muzzle length plays a role in people’s preference for dogs’ aesthetics. For future research, it would be worthwhile to use only flat-faced breeds with different muzzle lengths to reveal whether enthusiasts of flat-faced breeds still prefer extremely short muzzle lengths even if they are aware of the health risks.

A further main limitation of our study lies in the use of convenience sampling, which can lead to self-selection bias^113,114^. Individuals for whom their dog plays a central role in their life and who are interested in canine-related research are more likely to participate in such questionnaire studies-a trend we are also experiencing in behaviour studies. As common in similar studies on convenience samples^8,48,69,74,79,84,87,93,94^, the majority of respondents were women and owners of the animal species in question.

It is likely that our questionnaire mostly reached individuals familiar with the research activities of the Family Dog Project (https://ethology.elte.hu/Family_Dog_Project), as the study was primarily advertised on platforms associated with it. As a result, the sample likely consisted of respondents who were relatively well-informed about dogs. To balance pre-existing attitudes towards brachycephalic dogs, the questionnaire was subsequently advertised in Facebook groups dedicated to brachycephalic breeds, as it proved particularly challenging to reach enthusiasts of these dogs. Therefore, this subgroup may have primarily included individuals less familiar with the Family Dog Project and canine research.

Last but not least, our sample included diverse nationalities (although all countries represented have been shaped by Western European culture), with the majority of respondents holding Hungarian nationality (57.8%). The next most represented regions were North America (13.5%), German-speaking countries (6.6%), and the United Kingdom (5.6%)—all regions where canine research, including that on brachycephalic dogs, is particularly prominent. Thus, the results may be confined to their cultural backgrounds. However, to enable meaningful comparisons across subtle cultural differences, a larger and more internationally balanced sample would be required. Achieving this would require collaborative efforts among research laboratories worldwide, as most respondents typically originate from the country where the study is conducted^48,84^.

## Conclusion

The main novelties of our study lie in empirically demonstrating the role of dogs’ muzzle length in aesthetic preferences and exploring how this preference relates to psychological and social factors, thereby shedding light on a potential driver of the brachycephalic welfare crisis.

1. **Using a new method**,** we investigated the role of a single morphological trait—muzzle length—while keeping all other morphological traits constant.** Previous studies examining human preferences for brachycephalic dogs have examined breed choice, making it challenging to disentangle the role of muzzle length from other breed-specific traits (e.g., body size, coat type, ear shape). Our study was the first to experimentally investigate the role of muzzle length in aesthetic preferences, using digitally morphed images of the same dogs in which only muzzle length was varied while all other morphological traits were kept constant.
2. **Shorter muzzles are generally preferred**,** regardless of breed.** Our controlled approach demonstrated that this preference holds true independent of breed or other morphological traits. This suggests that human aesthetic preferences may contribute to selection pressures favouring shorter muzzles in companion animals, potentially driving both the trend toward decreasing skull length in modern dog breeds and the ongoing brachycephalic welfare crisis.
3. **Psychological and social factors are linked to the preference for shorter muzzle length.** While some prior studies have examined attitudes toward brachycephalic breeds, our work went further by showing that preferences for shorter muzzles correlate with specific human factors, including demographics, personality traits, motivations for dog ownership (e.g., prioritisation of aesthetic traits), and awareness of health issues. This integrative approach helps explain why public awareness campaigns alone may be insufficient to change demand for brachycephalic breeds.
4. **An interplay between pre-existing attitudes towards brachycephalic dogs and other human factors also contributes to shaping preferences for shorter muzzle lengths.** While health awareness is negatively associated with a preference for shorter muzzles among respondents without established attitudes towards brachycephalic dogs, this association does not hold once a positive attitude has developed. In the former group, a preference for shorter muzzles is positively linked to perceiving dogs as childlike, implying that individuals who seek child substitutes—perhaps unconsciously—are more likely to become prospective owners of brachycephalic breeds.

Based on our results, we suggest that educational campaigns for prospective owners should focus on prevention without needing to confront entrenched beliefs, whereas those for current brachycephalic dog enthusiasts must directly challenge misconceptions by clearly communicating the links between exaggerated anatomical features and associated health and welfare risks. Since enthusiasts often ignore factual information about brachycephalic dogs’ health issues^81,82,85^, future efforts—especially for non‑specialists—should bridge this knowledge gap to support more informed breed choices. Personality traits may also influence aesthetic preferences, potentially reflecting individual attitudes towards social norms, which creates an additional barrier to welfare-improving educational interventions. Further research should explore the influence of media, social media, and advertisements on the growing popularity of brachycephalic breeds. Individuals lacking professional expertise may be especially susceptible to popular culture portrayals of short-muzzled dogs as cute and attractive with insufficient attention paid to health-related concerns. It would also be valuable to investigate whether brachycephalic dog enthusiasts are selectively ignorant only of health issues related to brachycephalic breeds, or of canine health problems more broadly.

## Supplementary Information

Below is the link to the electronic supplementary material.

Supplementary Material 1

Supplementary Material 2

Supplementary Material 3

## Data Availability

Data is provided within the supplementary information files.
