# Supplementary material for: Selective attraction to shorter muzzles in dogs is a hidden driver of the brachycephalic welfare crisis
Source: Sci Rep. 2025 Oct 6;15:34699. doi: 10.1038/s41598-025-16562-9 (PMC12501307; doi:10.1038/s41598-025-16562-9)
Supplement: Supplementary file 1 — Supplementary Material 1 [file 41598_2025_16562_MOESM1_ESM.pdf]

Selective attraction to shorter muzzles in dogs is a hidden driver of the brachycephalic welfare crisis

Zsófia Bognár[1,2*], Yuri Kawaguchi[3], Koyo Nakamura[4,5,6], Enikő Kubinyi[1,2,7]

[1] MTA-ELTE Lendület "Momentum" Companion Animal Research Group, Budapest, Hungary

[2] Department of Ethology, ELTE Eötvös Loránd University, Budapest, Hungary

[3] School of Social Sciences, Nottingham Trent University, Nottingham, England

[4] Faculty of Science and Engineering, Waseda University, Tokyo, Japan

[5] Japan Society for the Promotion of Science (JSPS), Tokyo, Japan

[6] Faculty of Psychology, Department of Cognition, Emotion, and Methods in Psychology, University of Vienna, Vienna, Austria

[7] ELTE NAP Canine Brain Research Group, Budapest, Hungary

[*] Corresponding author. *E-mail address:* zsofia.bognar@ttk.elte.hu, *Postal address*: Eötvös Loránd University, Pázmány Péter sétány 1/c, 1117, Budapest, Hungary

Questionnaire

36 questions of the questionnaire. The questionnaire was built on the Google Forms platform.

Question 1 – I understand that my data will be used anonymously as part of the research database and I agree to join. * required

a) Yes
b) No *(Skip to questionnaire submission)*

DOG-RELATED EXPERIENCE

Question 2 – Have you ever lived with a dog? * required

a) Yes, and I was involved in its care
b) Yes, and I was not involved in its care
c) No *(Skip question 4)*

Question 3 – Do you have professional experience with dogs? * required

a) Yes, veterinary (veterinarian, assistant)
b) Yes, other (dog trainer, beautician, shelter worker/volunteer, researcher, etc.)
c) No

YOUR DOG

Question 4 – If you have ever lived with a dog, please describe its appearance or breed!

Free text

YOU AND THE DOGS

Question 5 – What role fits your ideal dog? * required

	disagree strongly	disagree a little	neither agree nor disagree	agree a little	agree strongly
Felow worker					
Pet					
Friend					
Family member					
Child					
A dog is more important to me than any other person					

Question 6 – Which traits are important for you in a dog? * required

	not important	less important	important a bit	very important
My ideal dog is safe with children				
My ideal dog shows affection toward me				
My ideal dog is fully housetrained				
My ideal dog is physically healthy				
My ideal dog lives until he/she is at least 10 years old				
My ideal dog is beautiful				
My ideal dog has low exercise requirements				
My ideal dog has a humorous personality				

Question 7 – What causes (or would cause) a pleasure, a good feeling for you in keeping a dog? * required

	disagree strongly	disagree a little	neither agree nor disagree	agree a little	agree strongly
Stroking, contact					
Providing company (e.g., reduces loneliness)					
Security, watchdog					
Teaching, training, sports					
Unconditional love					
Taking care of someone					

Development of rules, control, decision making					
Contact with other people (e.g., other dog owners)					
Dog walking					
Admire the dog's appearance					
Taking pictures of the dog					
Acknowledging by others that the dog is beautiful/cute					
Entertainment/play					

DOGS' APPEARANCE

Question 8 – Please describe if you have a favourite dog breed or appearance you particularly LIKE in a dog (e.g. coat, stature etc.)! If not, leave the field blank.

Free text

Question 9 – Please describe if you have a dog breed or appearance you particularly DISLIKE in a dog (e.g. coat, stature etc.)! If not, leave the field blank.

Free text

EVALUATING DOG PHOTOS

Question 10 – Photo of dog A * required

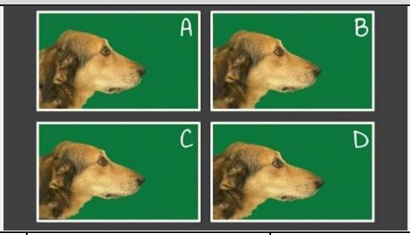

Which one is the most attractive?	A	B	C	D

Question 11 – Photo of dog B * required

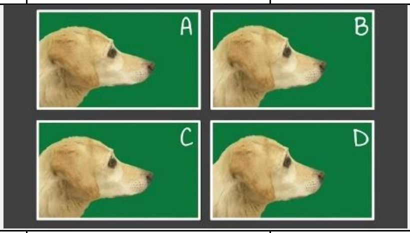

Which one is the most attractive?	A	B	C	D

Question 12 – Photo of dog C * required

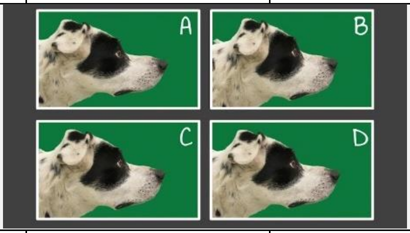

Which one is the most attractive?	A	B	C	D

Question 13 – Photo of dog D * required

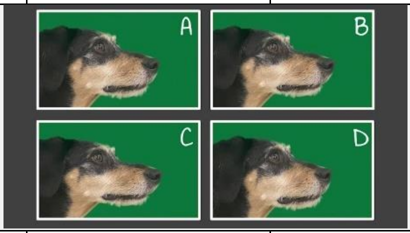

Which one is the most attractive?	A	B	C	D

**Question 14 –** Photo of dog E * required			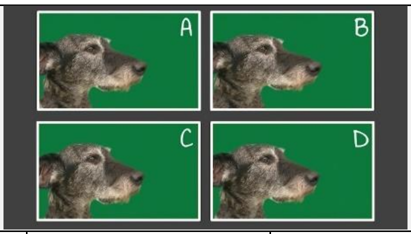	
Which one is the most attractive?	A	B	C	D
**Question 15 –** Photo of dog F * required			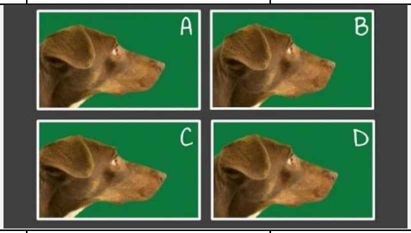	
Which one is the most attractive?	A	B	C	D
**Question 16 –** Photo of dog G * required			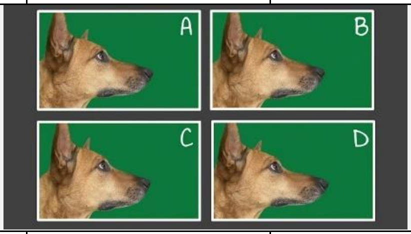	
Which one is the most attractive?	A	B	C	D
**Question 17 –** Photo of dog H * required			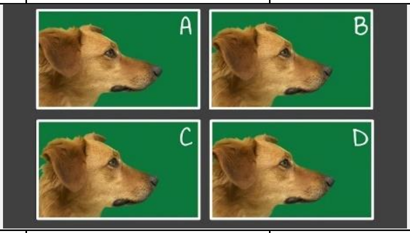	
Which one is the most attractive?	A	B	C	D
**Question 18 –** Photo of dog I * required			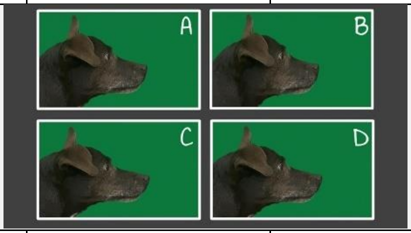	
Which one is the most attractive?	A	B	C	D
**Question 19 –** Photo of dog J * required			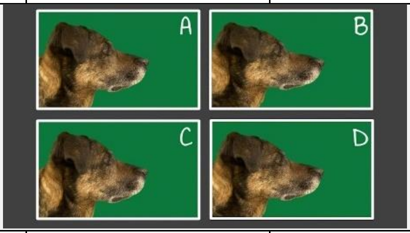	
Which one is the most attractive?	A	B	C	D

**Question 20 –** Photo of dog K * required	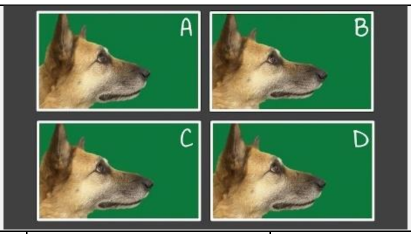			
Which one is the most attractive?	A	B	C	D
**Question 21 –** Photo of dog L * required	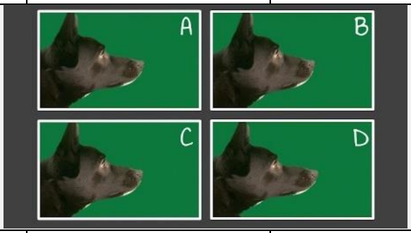			
Which one is the most attractive?	A	B	C	D
**Question 22 –** Photo of dog M * required	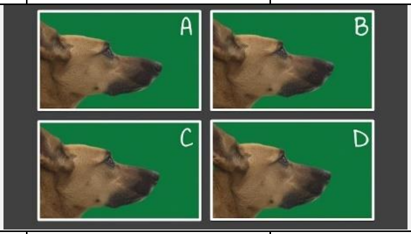			
Which one is the most attractive?	A	B	C	D
**Question 23 –** Photo of dog N * required	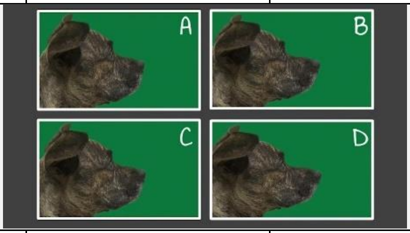			
Which one is the most attractive?	A	B	C	D

DOGS' HEALTH QUIZ

Question 24 – Please guess which breed(s) have a high risk of the following diseases in your opinion! You can mark more than one per line/column, and you can leave it blank, too.
Please do not use the internet to find answers. It is not important to give a good answer. We want to assess the extent to which diseases are in public knowledge.

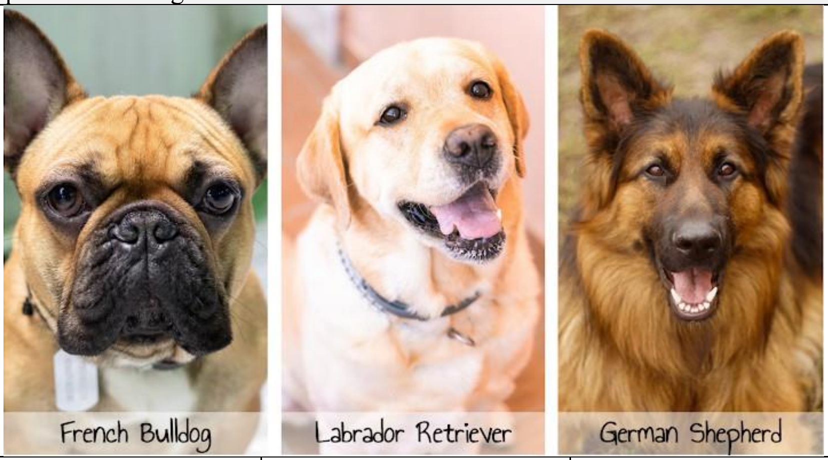

	French Bulldog	Labrador Retriever	German Shepherd
Dystocia			
Allergic skin diseases			
Breathing difficulties			
Corneal ulceration			

Obesity			
Abnormal teeth			
Joint diseases			

BASIC INFORMATION

Question 25 – Your gender * required

a) female
b) male
c) other

Question 26 – Your age (years) – We ask it because you may misspell your year of birth * required

Free text

Question 27 – Your year of birth – We ask it because you may misspell your age * required

Free text

Question 28 – What is your nationality? * required

Free text

Question 29 – Where is your place of residence (where you spend most of the week)? * required

a) Large metropolitan area (population size is above 1.5 milion)
b) Metropolitan area (population size is between 500.000 and 1.5 milion)
c) Medium-size urban area (population size is between 200.000 and 500.000)
d) Small urban area (population size is below 200.000)
e) Rural area

EDUCATION

Question 30 – Your highest level of education * required

a) Early childhood education
b) Primary education
c) Lower secondary education
d) Upper secondary education
e) Post-secondary non-tertiary education
f) Short-cycle tertiary education
g) Bachelor's or equivalent level
h) Master's or equivalent level
i) Doctoral or equivalent level

Question 31 – Did you receive education (higher than basic level) in health or natural science? * required

a) Yes
b) No

RELATIONSHIP WITH CHILDREN

Question 32 – How many children of yours were born? * required

Free text

Question 33 – Do you want (more) children now or in the future? * required

a) No
b) Yes
c) I do not know

Question 34 – Do you currently take care of a child under the age of 6 at least once a week? * required

a) No
b) Yes, but there are no children under the age of 6 in my household
c) Yes, and there are children under the age of 6 in my household

YOUR PERSONALITY

Question 35 – Here are a number of characteristics that may or may not apply to you. Please indicate the extent to which you agree or disagree with the statements! I see myself as someone who... * required

	disagree strongly	disagree a little	neither agree nor disagree	agree a little	agree strongly
is talkative					
tends to find fault with others					
does a thorough job					
is depressed, blue					
is original, comes up with new ideas					
is reserved					
is helpful and unselfish with others					
can be					

somewhat careless					
is relaxed, handles stress well					
is curious about many different things					
is full of energy					
starts quarrels with others					
is a reliable worker					
can be tense					
is ingenious, a deep thinker					
generates a lot of enthusiasm					
has a forgiving nature					
tends to be disorganized					
worries a lot					
has an active imagination					
tends to be quiet					
is generally trusting					
tends to be lazy					
is emotionally stable, not easily upset					
is inventive					
has an assertive personality					
can be cold and aloof					
perseveres until the task is finished					
can be moody					
values artistic, aesthetic experiences					
is sometimes shy, inhibited					
is considerate and kind to almost everyone					
does things efficiently					
remains calm in tense situations					
prefers work that is routine					
is outgoing, sociable					
is sometimes rude to others					
makes plans and follows through with them					
gets nervous easily					
likes to reflect, play with ideas					

has few artistic interests					
likes to cooperate with others					
is easily distracted					
is sophisticated in art, music, or literature					

END OF THE QUESTIONNAIRE

You will need a unique code to view the results of the Personality Test. If you don't want to view your results, just skip this part.

This is a guide, on how to generate a unique code:

The five-character unique code consists of:
1) the first letter of the month of birth (e.g. March -> m)
2) the last digit of your birthday (e.g. 12 -> 2)
3) the first letter of your mother's birth name (e.g. Smith -> s)
4) the second letter of your mother's first name (e.g. Rachel -> a)
5) the first letter of your father's first name (e.g. John -> j)

These are written in all lowercase letters, without spaces, to create your personal code. In the above case, the unique code will be m2saj

Question 36 – Unique code
A link will be available at the end of the questionnaire where you can view your results. Please write down your unique code because you will need it to see the results! If you do not want to see your results, leave the field blank.

Free text
