# Supplementary material for: Selective attraction to shorter muzzles in dogs is a hidden driver of the brachycephalic welfare crisis
Source: Sci Rep. 2025 Oct 6;15:34699. doi: 10.1038/s41598-025-16562-9 (PMC12501307; doi:10.1038/s41598-025-16562-9)
Supplement: Supplementary file 2 — Supplementary Material 2 [file 41598_2025_16562_MOESM2_ESM.pdf]

Selective attraction to shorter muzzles in dogs is a hidden driver of the brachycephalic welfare crisis

Zsófia Bognár[1,2*], Yuri Kawaguchi[3], Koyo Nakamura[4,5,6], Enikő Kubinyi[1,2,7]

[1] MTA-ELTE Lendület "Momentum" Companion Animal Research Group, Budapest, Hungary

[2] Department of Ethology, ELTE Eötvös Loránd University, Budapest, Hungary

[3] School of Social Sciences, Nottingham Trent University, Nottingham, England

[4] Faculty of Science and Engineering, Waseda University, Tokyo, Japan

[5] Japan Society for the Promotion of Science (JSPS), Tokyo, Japan

[6] Faculty of Psychology, Department of Cognition, Emotion, and Methods in Psychology, University of Vienna, Vienna, Austria

[7] ELTE NAP Canine Brain Research Group, Budapest, Hungary

[*] Corresponding author. *E-mail address:* bognarzsof@gmail.com, *Postal address*: Eötvös Loránd University, Pázmány Péter sétány 1/c, 1117, Budapest, Hungary

Additional information

Supplementary Table 1 – Muzzle length/head shape preference stimuli, Relative Unnaturalness Rank, choices by respondents, and Kendall's rank correlations. The excluded photo quadruplets are highlighted with grey.

Photo	Photo ID	Muzzle	Relative Unnaturalness Rank	Choice by respondents (frequency)	Kendall's rank correlation
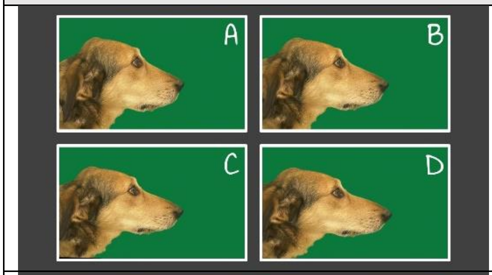	A	+15%	0.167	122	tau = -0.33, p = 0.750
		+5%	0	263	
		-5%	0.208	234	
		-15%	0.500	161	
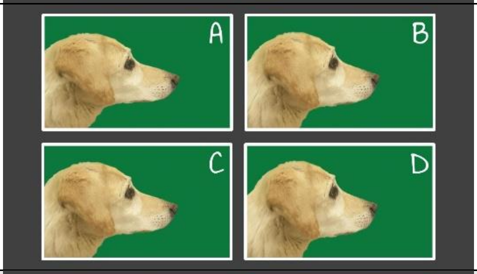	B	+15%	0.458	49	tau = -0.18, p = 0.718
		+5%	0	138	
		-5%	0.083	345	
		-15%	0.083	248	
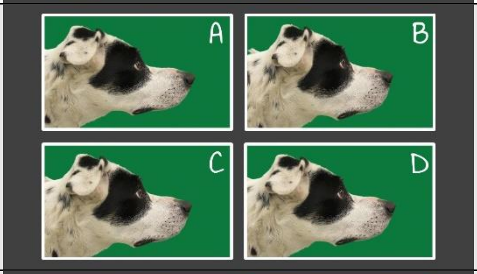	C	+15%	0.667	37	tau = -1.00, p = 0.083
		+5%	0.583	81	
		-5%	0.292	215	
		-15%	0	447	
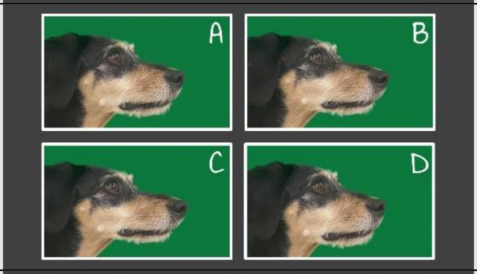	D	+15%	0.750	22	tau = -1.00, p = 0.083
		+5%	0.375	87	
		-5%	0.167	224	
		-15%	0	447	
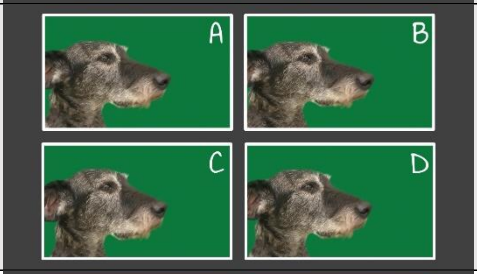	E	+15%	0.583	65	tau = -1.00, p = 0.083
		+5%	0.167	132	
		-5%	0.042	233	
		-15%	0	350	
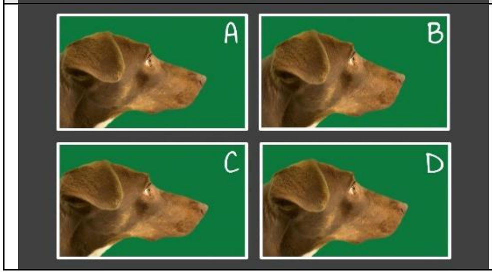	F	+15%	0.458	106	tau = -0.33, p = 0.750
		+5%	0	222	
		-5%	0.333	232	
		-15%	0.250	220	

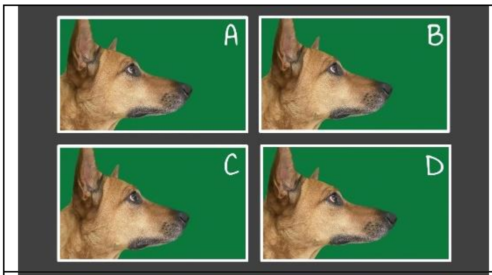	G	+15%	0.583	30	tau = -0.18, p = 0.718
		+5%	0	139	
		-5%	0.083	416	
		-15%	0	195	
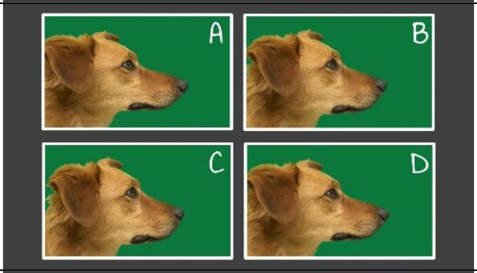	H	+15%	0.250	45	tau = -0.33, p = 0.750
		+5%	0.542	111	
		-5%	0.167	386	
		-15%	0	238	
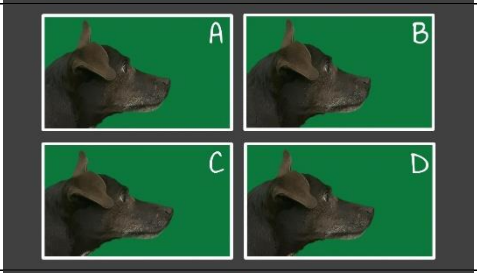	I	+15%	0.458	88	tau = 0.00, p = 1.000
		+5%	0	218	
		-5%	0.292	240	
		-15%	0.042	234	
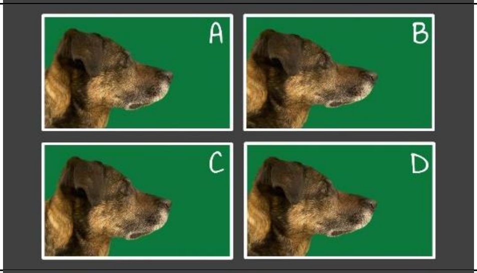	J	+15%	0.250	61	tau = -0.67, p = 0.333
		+5%	0.292	125	
		-5%	0	334	
		-15%	0.042	260	
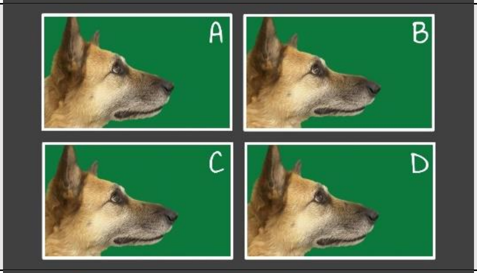	K	+15%	0.750	71	tau = -0.91, p = 0.071
		+5%	0	187	
		-5%	0	341	
		-15%	0.083	181	
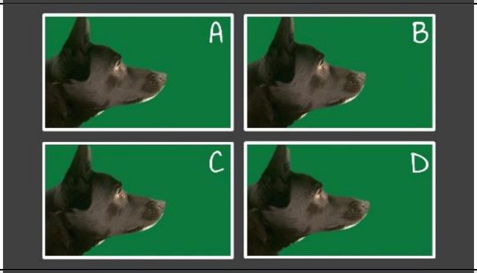	L	+15%	0.208	55	tau = -0.24, p = 0.655
		+5%	0.208	160	
		-5%	0	269	
		-15%	0.208	296	
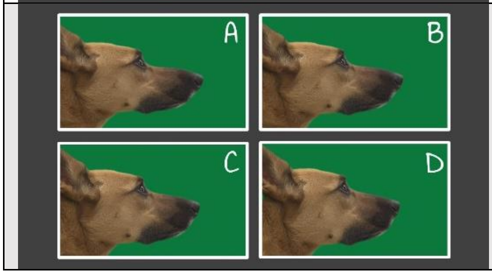	M	+15%	1	31	tau = -1.00, p = 0.083
		+5%	0.458	123	
		-5%	0.417	257	
		-15%	0	369	

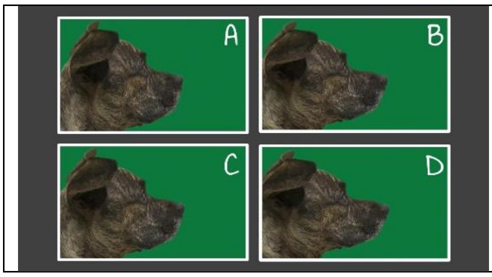	N	+15%	0.375	145	tau = 0.40, p = 0.444
		+5%	0.458	261	
		-5%	0	187	
		-15%	0.375	187	

Supplementary Table 2 – The characteristics of respondents.

1) Demography	
Residence (spend most of the time per week)	large metropolitan area: 32.9%<br>metropolitan area: 8.3%<br>medium-sized urban area: 14.7%<br>small urban area: 21.4%<br>rural area: 22.8%<br>*Metropolitan area & medium-sized urban area were grouped together for analyses as* urban area: 22.9%
Highest level of education	early childhood education: 0.0%<br>primary education: 0.0%<br>lower secondary education: 0.3%<br>upper secondary education: 9.2%<br>post-secondary non-tertiary education: 4.7%<br>short-cycle tertiary education: 9.2%<br>bachelor's or equivalent level: 34.7%<br>master's or equivalent level: 33.0%<br>doctoral or equivalent level: 8.9%<br>*Levels lower than university levels were grouped together for analyses as* non-university level: 23.3%
Dog-related professional expertise	Veterinary (veterinarian, assistant): 6.6%<br>Other dog-related expertise (dog trainer, dog groomer, shelter worker/volunteer, researcher, etc.): 38.3%<br>No: 55.2%<br>*Veterinary & other dog-related expertise were grouped together for analyses as* dog-related expertise: 44.8%
Advanced-level education in health or natural science	No: 53.7%<br>Yes: 46.3%
How many children were born	0: 64.2%<br>1: 12.7%<br>2: 17.6%<br>3: 4.1%<br>4: 1.3%<br>5: 0.1%
Want (more) children	Yes: 19.5%<br>No: 66.2%<br>Don't know: 14.3%
Currently taking care of a child under the age of 6 at least once a week	Yes, own children: 5.1%<br>Yes, not own children: 7.3%<br>No: 87.5%
2) Personality (Big Five)	
Extraversion (1-5)	1.1-5.0 scores (median ± SD: 3.4 ± 0.8)
Agreeableness (1-5)	1.2-5.0 scores (median ± SD: 3.7 ± 0.6)
Conscientiousness (1-5)	1.8-5.0 scores (median ± SD: 3.7 ± 0.6)
Neuroticism (1-5)	1.0-4.9 scores (median ± SD: 3.0 ± 0.8)
Openness (1-5)	1.6-5.0 scores (median ± SD: 3.7 ± 0.6)
3) Knowledge of brachycephalic dogs' health problems	
Number of health problems associated with brachycephalism (0-7)	0: 0.7%<br>1: 0.9%<br>2: 3.3%<br>3: 7.7%<br>4: 16.9%<br>5: 23.3%<br>6: 23.1%<br>7: 24.1%

Supplementary Table 3 – Proportions of responses (N = 763) regarding a hypothetical ideal companion dog's traits, attributed roles, and aspects of dog ownership that respondents find rewarding.

1) Traits of the ideal dog	not important	less important	important a bit	very important
Safe with children	8.0%	13.2%	29.1%	49.7%
Shows affection towards the owner	0.5%	2.8%	24.1%	72.6%
Fully housetrained	2.4%	6.4%	26.7%	64.5%
Physically healthy	1.8%	5.6%	24.9%	67.6%
Lives until he/she is at least 10 years old	3.0%	8.1%	21.0%	67.9%
Beautiful	21.2%	35.0%	27.8%	16.0%
Low exercise requirements	39.7%	38.1%	18.9%	3.3%
Humorous personality	8.9%	22.5%	37.4%	31.2%

2) Role of the ideal dog	disagree strongly	disagree a little	neither agree nor disagree	agree a little	agree strongly
Fellow worker	22.5%	22.1%	23.2%	18.3%	13.8%
Pet	9.3%	7.6%	19.3%	22.8%	41.0%
Friend	1.0%	2.1%	5.9%	21.9%	69.1%
Family member	0.8%	0.9%	4.2%	13.6%	80.5%
Child	27.4%	19.1%	25.3%	12.7%	15.5%
A dog is more important to me than any other person	16.6%	17.8%	24.6%	26.5%	14.4%

3) Pleasure in dog keeping	disagree strongly	disagree a little	neither agree nor disagree	agree a little	agree strongly
Stroking, contact	0.5%	0.5%	5.9%	17.4%	75.6%
Providing company (e.g., reduces loneliness)	0.9%	2.0%	7.6%	22.9%	66.6%
Security, watchdog	19.3%	19.9%	26.2%	23.3%	11.3%
Teaching, training, sports	5.1%	9.3%	21.0%	29.4%	35.3%
Unconditional love	2.5%	2.2%	7.2%	21.2%	66.8%
Taking care of someone	3.3%	8.0%	18.7%	30.5%	39.4%
Development of rules, control, decision making	20.1%	20.8%	29.0%	19.3%	10.9%
Contact with other people (e.g., other dog owners)	14.2%	20.7%	28.8%	25.7%	10.6%
Dog walking	2.6%	6.8%	15.5%	32.4%	42.7%
Admire the dog's appearance	9.4%	12.1%	21.4%	29.9%	27.3%
Taking pictures of the dog	13.9%	15.2%	24.1%	25.8%	21.0%
Acknowledging by others that the dog is beautiful/cute	29.6%	22.7%	26.1%	13.4%	8.3%
Entertainment/play	3.0%	4.8%	13.0%	32.1%	47.1%
